# Association between flower stalk elongation, an *Arabidopsis* developmental trait, and the subcellular location and movement dynamics of the nonstructural protein P3 of *Turnip mosaic virus*

**DOI:** 10.1111/mpp.12976

**Published:** 2020-08-01

**Authors:** Silvia López‐González, José Antonio Navarro, Luis F. Pacios, Papaiah Sardaru, Vicente Pallás, Flora Sánchez, Fernando Ponz

**Affiliations:** ^1^ Centro de Biotecnología y Genómica de Plantas (UPM‐INIA) Pozuelo de Alarcón Spain; ^2^ Instituto de Biología Molecular y Celular de Plantas (UPV‐CSIC), IBMCP Valencia Spain

**Keywords:** endoplasmic reticulum streaming, P3 protein, peripheral membrane protein, plant developmental traits, potyvirus, surface electrostatic potential, Turnip mosaic virus

## Abstract

Virus infections affect plant developmental traits but this aspect of the interaction has not been extensively studied so far. Two strains of *Turnip mosaic virus* differentially affect *Arabidopsis* development, especially flower stalk elongation, which allowed phenotypical, cellular, and molecular characterization of the viral determinant, the P3 protein. Transiently expressed wild‐type green fluorescent protein‐tagged P3 proteins of both strains and selected mutants of them revealed important differences in their behaviour as endoplasmic reticulum (ER)‐associated peripheral proteins flowing along the reticulum, forming punctate accumulations. Three‐dimensional (3D) model structures of all expressed P3 proteins were computationally constructed through I‐TASSER protein structure predictions, which were used to compute protein surfaces and map electrostatic potentials to characterize the effect of amino acid changes on features related to protein interactions and to phenotypical and subcellular results. The amino acid at position 279 was the main determinant affecting stalk development. It also determined the speed of ER‐flow of the expressed proteins and their final location. A marked change in the protein surface electrostatic potential correlated with changes in subcellular location. One single amino acid in the P3 viral protein determines all the analysed differential characteristics between strains differentially affecting flower stalk development. A model proposing a role of the protein in the intracellular movement of the viral replication complex, in association with the viral 6K2 protein, is proposed. The type of association between both viral proteins could differ between the strains.

## Graphical abstract

Two strains of *Turnip mosaic virus* differentially affect *Arabidopsis* flower stalk elongation. A single amino acid change in the viral P3 protein interchanges plant phenotypes and the subcellular behaviour of the protein.

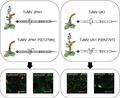

## 1 INTRODUCTION

Plant viruses can alter the development of infected plants. In addition to symptoms traditionally associated with viral infections such as leaf mosaics, yellows, or a general dwarfism, viruses often induce alterations in typical plant traits like androsterility, shape alteration in the temporal evolution of different organs, or the lack of development of reproductive organs. This development‐related aspect has received less attention compared with the study of defence responses, for example. We have recently reviewed the intimate relationship between virus infections and alterations in developmental traits, and discussed the different viral and plant components identified so far in this complex relationship (Sánchez and Ponz, 2018).

An appropriate model system for the study of developmental alterations is the pathosystem formed by the potyvirus *Turnip mosaic virus* (TuMV) and *Arabidopsis thaliana*. TuMV encapsidates a single genomic RNA encoding all viral proteins, whose expression is mostly mediated by proteolytic processing of a precursor polyprotein (Ivanov *et al*., 2014; Revers and García, 2015), plus two additional fusion proteins, P3N‐PIPO (Chung *et al*., 2008) and P3N‐ALT (Hagiwara‐Komoda *et al*., 2016). Two isolates/strains of TuMV (UK 1 and JPN 1) alter development differentially (Sánchez *et al*., 2015), and TuMV infections in *Arabidopsis* are also affected by the developmental stage of the plant (Lunello *et al*., 2007). A particularly dramatic change affects flower stalk elongation. Plants grown under long day conditions, inoculated with virus at developmental stage 1.08 (Boyes *et al*., 2001), were differentially affected: UK 1‐infected plants did not elongate a flower stalk, whereas JPN 1‐infected did. Other alterations in plant architecture were also found but this was the main difference, which has dramatic effects on the fertility and reproduction of the plants, depending on the infecting isolate.

The implication of virus‐encoded suppressors of RNA silencing (VSRs) as the main viral factors involved in developmental alterations has been raised (Kasschau *et al*., 2003; Chapman *et al*., 2004; Chellappan *et al*., 2005), although this view has been also questioned (Mlotshwa *et al*., 2005). In the case of stalk elongation of TuMV‐infected *Arabidopsis* plants, the major viral determinant is the protein P3, not a VSR (Sánchez *et al*., 2015). The determinant was mapped to the C‐terminal coding region of the cistron, out of the genomic region involved in the formation of the fusion proteins P3N‐PIPO and P3N‐ALT.

Not much is known about the role of P3 in potyviral infections, although different aspects of it have been covered. The protein has been described as a membrane protein (Eiamtanasate *et al*., 2007; Cui *et al*., 2010), a determinant of viral symptoms (Sáenz *et al*., 2000; Dallot *et al*., 2001; Desbiez *et al*., 2003; Jenner *et al*., 2003; Sánchez *et al*., 2015), of avirulence in resistance situations (Johansen *et al*., 2001; Hjulsager *et al*., 2002, 2006; Kim *et al*., 2010), and of host range (Suehiro *et al*., 2004; Tan *et al*., 2005; Salvador *et al*., 2008). An RNA element in the P3 cistron was found to affect viral replication and movement (Choi *et al*., 2005). In the case of TuMV P3, the protein has been identified as a determinant of the three responses mentioned (Jenner *et al*., 2002, 2003; Suehiro *et al*., 2004; Tan *et al*., 2005; Kim *et al*., 2010; Sánchez *et al*., 2015; Cui *et al*., 2017), and the relevance of its C‐terminal region has been highlighted (Suehiro *et al*., 2004; Tan *et al*., 2005; Cui *et al*., 2017). Potential interactions of P3 have been proposed for different potyviruses, and its subcellular location has also received attention. It has been proposed that P3 interacts (or has the capability to do so) with other viral proteins such as P1, HC‐Pro, CI, NIa, NIb, and VPg (Rodríguez‐Cerezo *et al*., 1993, 1997; Merits *et al*., 1999; Guo *et al*., 2001; Shen *et al*., 2010; Zilian and Maiss, 2011), or that no interactions take place with any of these proteins (Urcuqui‐Inchima *et al*., 1999). It was also proposed to interact with itself (Choi *et al*., 2000) and with no other viral protein (Kang *et al*., 2004). Very recently, the interaction of P3 with the fusion protein P3N‐PIPO has been shown for TuMV (Chai *et al*., 2020). Interactions with plant proteins have also been reported, including ADF‐2 (cofilin 2) (Lu *et al*., 2015), eEF1A (Luan *et al*., 2016), actin (Cui *et al*., 2010), RuBisCO (Lin *et al*., 2011), and RHP (Cui *et al*., 2018). Its subcellular location is controversial because it has been associated with the endoplasmic reticulum (ER) through its C‐terminal region (Eiamtanasate *et al*., 2007; Cui *et al*., 2010, 2017), Golgi vesicles through transmembrane domains (Eiamtanasate *et al*., 2007; Cui *et al*., 2010, 2017), nucleus (Langenberg and Zhang, 1997), and 6K2‐induced vesicles (Cui *et al*., 2010, 2017). This complex scenario requires clarification in order to understand P3 role(s) in infections.

Because P3 is the determinant for stalk elongation in TuMV‐infected *Arabidopsis* plants, we are interested in linking P3 subcellular behaviour with its impact on plant development through studies at the cellular level, including mutant analysis. The results obtained show differential subcellular behaviour of P3 between elongation‐allowing and elongation‐arresting TuMV strains. We also identify a mutant in a single amino acid position able to interconvert the infection phenotypes.

## 2 RESULTS

### 2.1 TuMV P3 as an ER‐associated peripheral membrane protein

TuMV P3, like other potyvirus P3 proteins (Eiamtanasate *et al*., 2007; Cui *et al*., 2010), is an ER‐associated protein (Cui *et al*., 2017). To relate this fact to the determination of flower stalk elongation, we comparatively studied transiently expressed UK 1 and JPN 1 P3 proteins, C‐terminally fused to the fluorescent tags green fluorescent protein (GFP) and cherry fluorescent protein (ChFP). The use of N‐terminal fusions was not advisable because the N‐terminal region of P3 is also part of the fusion proteins P3N‐PIPO (Chung *et al*., 2008) and P3N‐ALT (Hagiwara‐Komoda *et al*., 2016), both of which were discarded as determinants of stalk arrest (Sánchez *et al*., 2015). The following fusion constructs were generated: P3[UK1]‐GFP, P3[JPN1]‐GFP, P3[UK1]‐ChFP, and P3[JPN1]‐ChFP. They were agroinfiltrated in *Nicotiana benthamiana*, harvested 1–2 days postinoculation (dpi), and examined by confocal microscopy. Preliminary observations showed that the constructs providing the highest fluorescence were P3[UK1]‐GFP and P3[JPN1]‐GFP, so these were chosen for the analyses.

**FIGURE 1:**
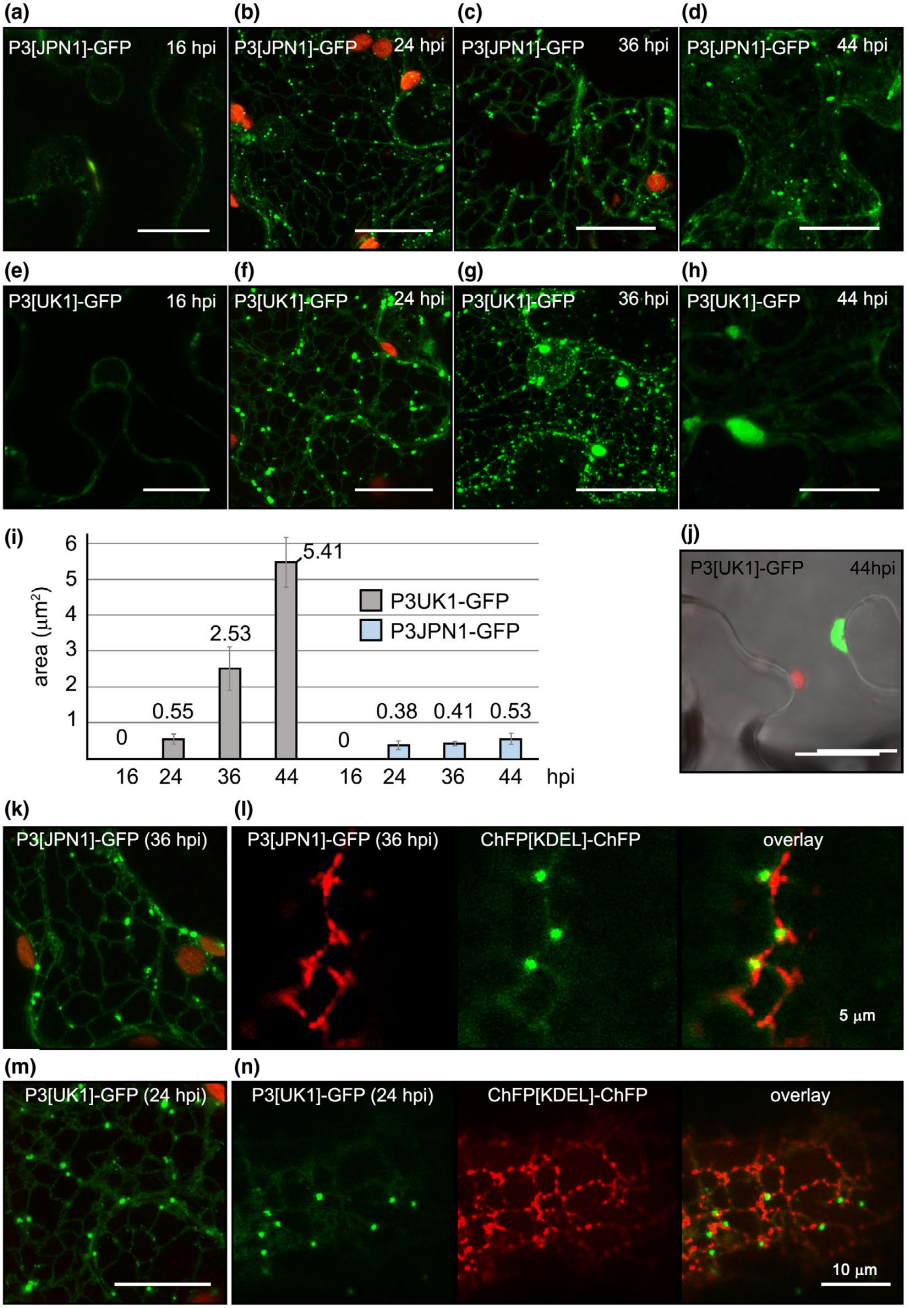
Time course of subcellular localization of *Nicotiana benthamiana* transiently expressed TuMV P3‐GFP. (a)–(d) P3[JPN1]‐GFP; (e)–(h) P3[UK1]‐GFP; (i) average sizes of P3[UK1]‐GFP and P3[JPN1]‐GFP punctate structures; (j): *z*‐series stack projection showing a large P3[UK1]‐GFP aggregate near the cell periphery at 44 hr postinoculation (hpi); (k), (m) enlarged view of P3[JPN1]‐GFP (k) and P3[UK1]‐GFP (m) fluorescent distribution pattern at 36 and 24 hpi, respectively; (l), (n): co‐expresssion of P3[JPN1]‐GFP (l) or P3[UK1]‐GFP (n) and the endoplasmic reticulum marker ChFP‐KDEL. Except for (a), (e), (l), and (n) (single‐scan images), the remaining images are *z*‐series stack projections. Except where indicated, scale bars are 20 µm

A time‐course study was performed (16, 24, 36, and 44 hr postinoculation, hpi). At 16 hpi only a diffuse and faint fluorescence was seen in all cells, not labelling any specific structure (Figure 1a,e). At 24 hpi, P3[JPN1]‐GFP led to the appearance of cytoplasmic discrete punctate structures (Figure 1b), sized 0.2–1.3 µm^2^ (average 0.38 µm^2^, Figure 1i), representing most of the fluorescence. The remaining fluorescence formed a faint reticulate structure. In contrast, P3[UK1]‐GFP strongly labelled a reticulate structure (Figure 1f), although its induced punctate accumulations were not significantly different in size compared to those induced by P3[JPN1]‐GFP (*q* = 1.72, *p* = .83). Most of them ranged from 0.2 to 1.5 µm^2^ (average 0.55 µm^2^, Figure 1i), although some of them (0.03%–0.04%) were considerably bigger than that, reaching sizes up to 7–8 µm^2^. At 36 hpi, both P3‐GFPs still labelled the cortical ER polygonal and tubular network. P3[JPN1]‐GFP punctate structures were slightly (but significantly, *q* = 17.57, *p* = .0001) bigger than at 24 hpi (average 0.41 µm^2^, Figure 1i), and the intensity of the ER‐associated fluorescence was slightly higher than at 36 hpi (Figure 1c). However, the fluorescence of P3[UK1]‐GFP started disappearing from the ER (ER:punctate structures fluorescence ratio 6.5 ± 2 at 24 hpi vs. 3.4 ± 1 at 36 hpi) and accumulating in significantly larger aggregates (average 2.53 µm^2^) than P3[UK1]‐GFP at 24 hpi (*q* = 16.80, *p* < .0001; Figure 1i), some of them (7%–10%) ranging from 3 to 30 µm^2^ (Figure 1g). At 44 hpi, the P3[JPN1]‐GFP distribution pattern was similar to that observed at 36 hpi (Figure 1d) with the small exception that punctate particles became slightly bigger (average 0.53 µm^2^, Figure 1i), reaching sizes similar to the 24 hpi P3[UK1]‐GFP particles (*q* = 38.22, *p* > .99). At this time, P3[UK1]‐GFP ER‐associated fluorescence faded and almost disappeared in some cells (ER:punctate structures fluorescence ratio 1.0 ± 0.4) (Figure 1h). In contrast, P3[UK1]‐GFP aggregates were larger than before (*q* = 19.99, *p* < .0001), reaching an average of 5.41 µm^2^ (Figure 1i). Nevertheless, 10–50 µm^2^ particles were in a proportion of 10%–20%. Two‐way analysis of variance (ANOVA) also showed statistical significance between P3 variants (*F* = 607.4, *p* < .0001), time (*F* = 227.0, *p* < .0001), and the interaction between P3 variants and time (*F* = 200.2, *p* < .0001), indicating that differences in size between P3[UK1]‐GFP and P3[JPN1]‐GFP aggregates increased over time. Visualization of these aggregates by *z*‐series stack projection and 360° 3D videos showed them as patches of different sizes accumulating at cortical regions close to the cell periphery (Figure 1j, Videos S1 and S2). Western blots at 44 hpi of total protein extracts from these leaves revealed that both P3‐GFP fusion proteins had the correct size (approximately 68 kDa) and similar accumulation levels (Figure S1).

To confirm that the fluorescent reticulate was ER, each fusion protein was co‐expressed together with a red ER‐luminal marker, ChFP‐KDEL (Serra‐Soriano *et al*., 2014) (Figure S2). At 24 hpi, the net in the P3[UK1]‐GFP agroinfiltrated plants overlapped with the ER marker (Pearson correlation coefficient [PCC] 0.799, Figure S2a–d). A similar image was obtained with P3[JPN1]‐GFP at 36 hpi (PCC 0.746, Figure S2e–h). Interestingly, cytoplasmic punctate accumulations clearly associated with the reticulum (see enlarged *z*‐series stack images in Figure 1k,m, 360° 3D Videos S3 and S4 and enlarged scans of P3‐GFP and ChFP‐KDEL co‐expression in Figure 1l,n).

**FIGURE 2:**
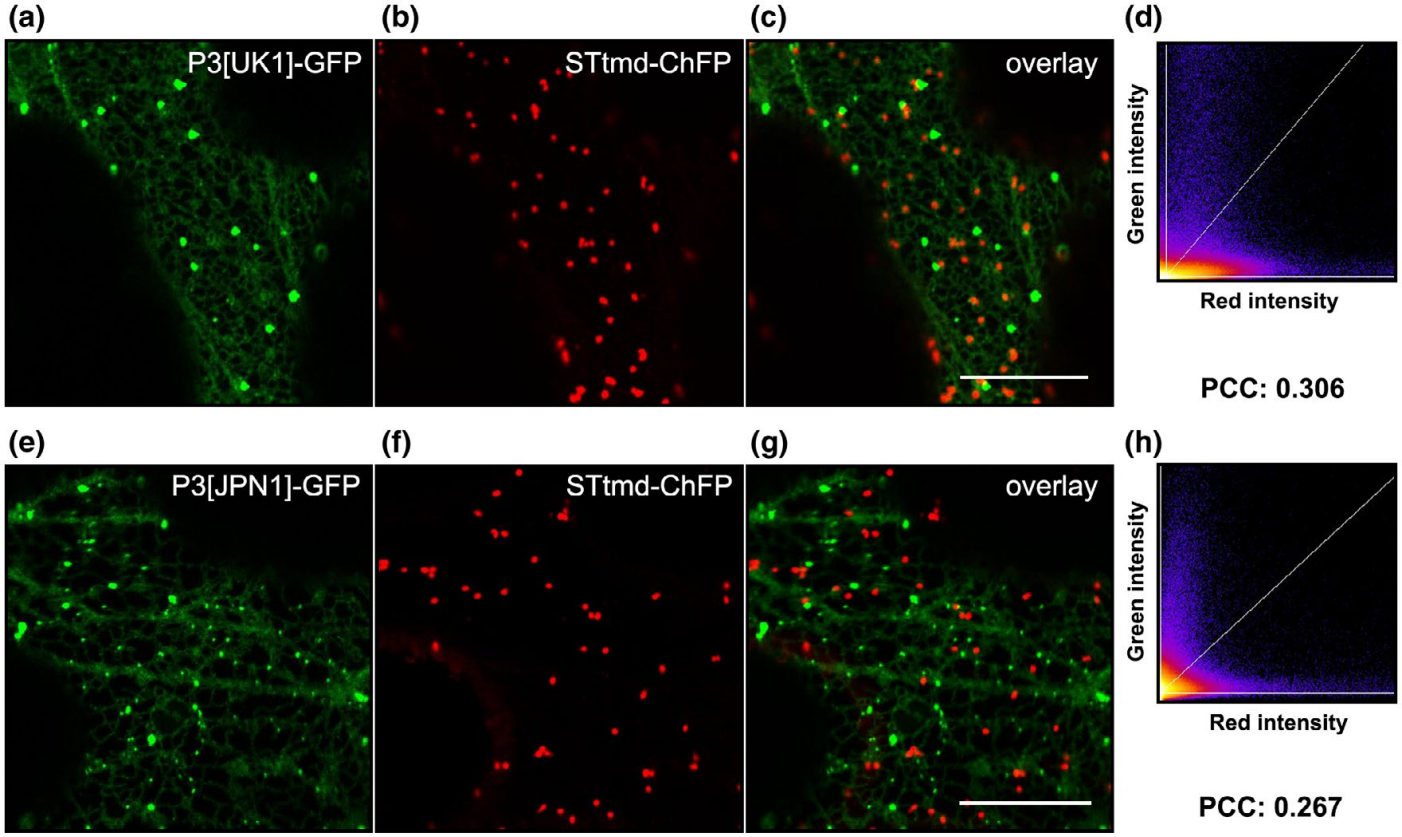
Confocal laser microscopy *z*‐series stack projection of epidermal cells co‐expressing P3[UK1]‐GFP (a) or P3[JPN1]‐GFP (e), and the Golgi marker STtmd‐ChFP (b) and (f). Overlay images of red and green channels (c) and (g). 2D histograms showing no correlation between pixel intensities over space of the two colour channels and Pearson correlation coefficient (PCC) values (d) and (h). Scale bars are 20 µm

*Tobacco etch virus* (TEV) P3 has been reported to form punctate inclusions in association with the Golgi apparatus (Cui *et al*., 2010). In our studies, P3 particles seemed to behave like they are associated with the Golgi apparatus because they were permanently associated with the ER, showing a dynamic movement (see next section). However, in contrast to the uniform size showed by the dictyosomes, P3‐GFP particles displayed a more heterogeneous size distribution within a single cell, changing with time. To address this point, both P3[UK1]‐GFP and P3[JPN1]‐GFP were separately co‐expressed with the Golgi marker STtmd‐ChFP (Serra‐Soriano *et al*., 2014). Neither P3‐GFP fusion protein co‐localized with STtmd‐ChFP (PCC: 0.3 for P3[UK1]‐GFP; 0.267 for P3[JPN1]‐GFP), confirming that transiently expressed TuMV P3‐GFPs associate with the ER but are not further exported to the Golgi apparatus (Figure 2).

**FIGURE 3:**
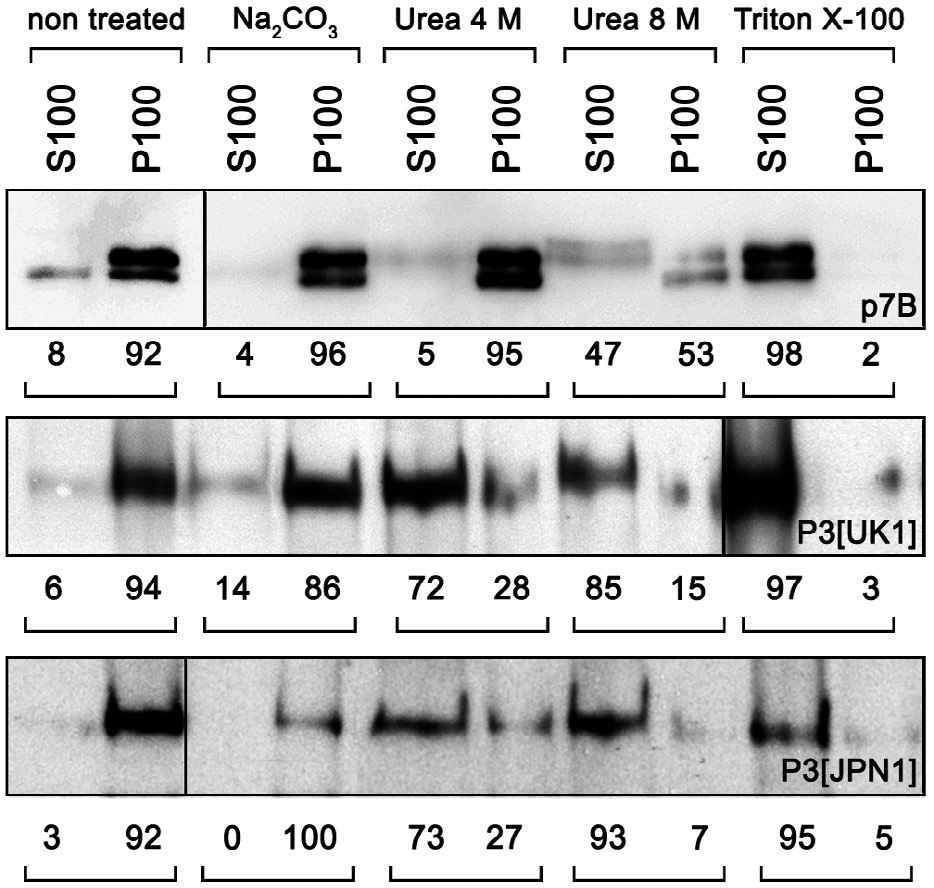
Peripheral endoplasmic reticulum (ER)‐membrane association of P3‐GFP proteins determined by differential treatments of the microsomal fraction. Extraction procedures differentiate luminal (Na_2_CO_3_), peripheral (urea), and integral (Triton X‐100) membrane proteins. The figure shows a western blot with anti‐GFP antibody. S100 and P100 refer to the 100,000 × g supernatant and pellet. Paired numbers correspond to signal intensity percentages showing the protein fraction distribution

TEV P3 was also predicted to contain two transmembrane domains (Cui *et al*., 2010), and thus to be an integral membrane protein. The characteristic of integral or peripheral protein was addressed by selective extraction procedures. The S100 and P100 fractions (soluble and microsomal, respectively) of agroinfiltrated leaf tissue were obtained and subjected to different treatments to release the soluble luminal proteins (Peremyslov *et al*., 2004), to dislodge proteins weakly or peripherally associated to membranes (Schaad *et al*., 1997), or to release integral proteins with a nonionic detergent (Triton X‐100). Western blots using an anti‐GFP antibody showed that the first treatment (0.1 M Na_2_CO_3_) did not affect P3 membrane association, indicating that P3 is not a luminal protein (Figure 3). In contrast, the Triton X‐100 treatment totally dislodged P3 proteins from the membranes. The presence of the GFP‐fused p7B protein of *Melon necrotic spot virus* (MNSV), a well‐known transmembrane protein of the ER/Golgi apparatus (GFP‐p7B) (Genovés *et al*., 2010; Serra‐Soriano *et al*., 2014), in the S100 fraction of the Triton X‐100 treatment confirmed that the extraction procedure separated soluble away from membrane proteins. However, urea treatments differentially affected both P3‐GFPs with respect to the GFP‐p7B. In 4 M urea, almost all GFP‐p7B remained in the P100 fraction (95%) while P3‐GFP was mostly recovered in the S100 fraction in both isolates (72% for P3[UK1]‐GFP and 73% for P3[JPN1]‐GFP), indicating that both P3‐GFPs are peripheral membrane proteins, rather than integral ones.

### 2.2 The ER‐movement of P3 particles is actomyosin‐dependent

Transiently expressed P3‐GFPs forming ER‐linked aggregates increasing in size suggested movement along the ER. To visualize the movement, videos were taken from agroinfiltrated tissues. At 1 dpi small and medium‐sized P3[UK1]‐GFP particles were highly ER‐mobile (Video S5 and particle tracking in Figure S3a). Some particles oscillated around the same ER location, but others were displaced fast. At 2 dpi, most of the fluorescence was in large immobile aggregates (Video S6). Some small and medium‐sized particles still remained, but the high mobility was lost. For P3[JPN1]‐GFP, the mobility of the particles was still high at 2 dpi (Video S7 and particle tracking in Figure S3b), similarly to the UK 1 protein at 1 dpi.

In plants, organelle and particle ER‐associated movement (ER streaming) is mostly recognized as actin‐dependent through myosin motors (Nebenfuhr *et al*., 1999; Ueda *et al*., 2010). We therefore used latrunculin B (Lat B), a well‐known actin microfilament disruptive agent (Holzinger and Blaas, 2016). LatB treatments require optimization. In our case, 1 µM Lat B treatments for 10 min did not affect ER structure, whereas 2 µM affected ER integrity, thus 1 µM was used in subsequent experiments. Treatment with 1 µM Lat B at 1 dpi (UK 1) or 2 dpi (JPN 1) stopped the advancement of ER‐associated aggregates, which just kept oscillating (Videos S8 and S9, respectively). Moreover, co‐expression of the P3‐GFPs with an actin microfilament marker (dsRFP‐Talin) showed their alignment along microfilaments (Figure S4a–f). This alignment was not so obvious when both P3‐GFPs were co‐expressed with a microtubule marker (αTubulin‐ChFP) (Figure S4g–l). Based only on this lack of co‐localization we cannot rule out the possible involvement of microtubules in P3‐GFP movement. However, treatments using colchicine, a microtubule disrupting agent, did not affect P3‐GFP movement (data not shown). Taken together, these results show that the ER‐associated movement of P3‐GFP proteins was dependent on the integrity of the actomyosin cytoskeleton.

### 2.3 P3 3D structural models predict differences between both isolates in C‐terminal regions

TEV P3 has been predicted to contain two transmembrane domains (TMDs), typical features of integral membrane proteins (Cui *et al*., 2010). TuMV P3 is a membrane peripheral protein, and is thus not very likely to contain TMDs. Nevertheless, exploratory searches with different computer programs to predict TMDs from sequence rendered TMDs for both TuMV P3 proteins (Figure S5). We therefore took a different approach to find out if there are TMDs in P3 by predicting full 3D structures. Conventional TMD prediction is based on sequence and its reliability depends on the similarity of the query sequence with sequences in the data sets used. In the case of TuMV P3 proteins, no experimental structures for homologous transmembrane proteins exist in the Protein Data Bank (PDB), thus the reliability of TMD prediction is limited and homology‐based 3D modelling is not possible. Consequently, we decided to apply I‐TASSER (Roy *et al*., 2010), a method ranking top in critical assessment of protein structure prediction experiments (Moult *et al*., 2014). I‐TASSER estimates the quality of predicted structural models through a confidence score (C‐score) that typically ranges from −5 to +2, with higher values indicating structural models with a higher confidence (Roy *et al*., 2010; Yang *et al*., 2014).

**FIGURE 4:**
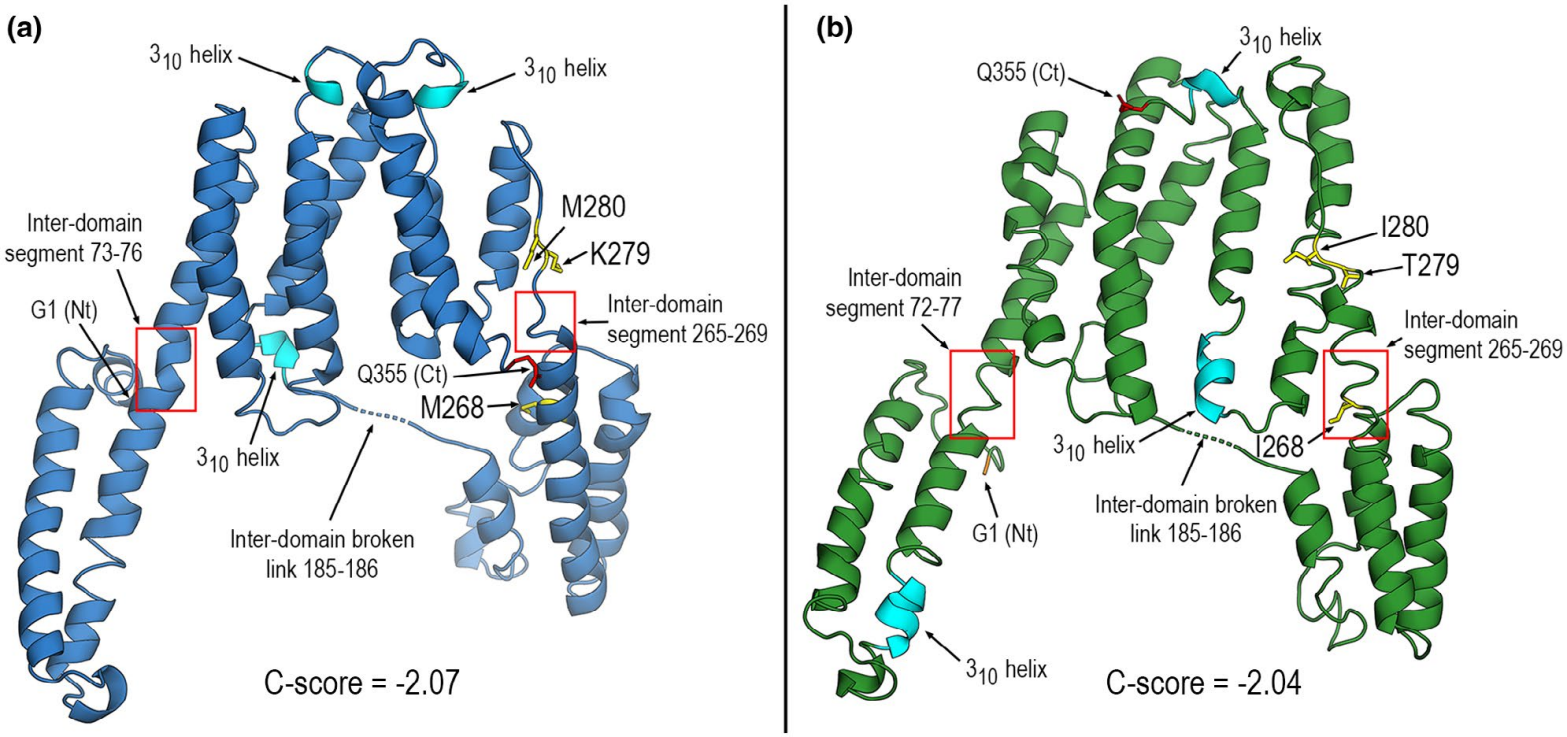
In silico modelled structures of P3 proteins. Ribbon diagrams of I‐TASSER predicted structures of P3[UK 1] (a) and P3[JPN 1] (b). Structural segments mentioned in the text, 3_10_ helices and both ends of structures are marked. Location in the structures of mutant positions 268, 279, and 280 are indicated. Side chains depicted as yellow sticks

C‐scores for 3D models of TuMV P3 were −2.07 (UK 1) and −2.04 (JPN 1), a medium level reliability. They are shown in Figure 4 with a consensus representation of subdomains. Secondary structure was identified with the Dictionary of Protein Secondary Structure (DSSP) (Kabsch and Sander, 1983; Touw *et al*., 2015), which is the PDB‐recommended standard method. Overall, the structural models for the 355‐residue P3 proteins revealed similar architectures with two differentiated large domains: a core helical bundle (Figure 4, upper part of structures) and two separated helical subdomains (Figure 4, lower segments at both ends of upper parts). The core domain spans the segment between positions c.75 and c.265, together with the C‐terminal sequence c.270–355 (Figure 4, right‐hand sides of upper parts). With this arrangement, the two separated helical substructures at both ends of the core might be dubbed “N‐terminal” (approximately the first 70 residues at the N‐terminus) and “C‐terminal” (segment c.200–270) subdomains. Although not sharply defined, these two subdomains are linked to the core domain through (a) two short segments around residues 72–77 and 265–269, and (b) a coil segment spanning approximately residues 180–190. With I‐TASSER, unconnected subdomains may appear at times when far apart in space. This is the case with some models of both P3s around positions 185 and 186. This artefactual result is a minor issue, not seriously affecting protein surfaces. The gap is reconstructed by default in most molecular graphics software.

Whereas the N‐terminal subdomain shows a rather similar motif of two helices with similar lengths, the C‐terminal subdomain displays a different number, spatial arrangement, and length of its α‐helices. Locations in the model structures of the mutations discussed below are indicated in Figure 4.

I‐TASSER performs alignments in the PDB searching for proteins structurally close to the predicted model. Top 10 proteins having the closest similarity to the model are ranked by TM‐score, a (0–1) metric where 1 indicates perfect match (Zhang and Skolnick, 2004). For the P3 models, only three proteins were found to have significantly similar structure (TM‐score > 0.660): two crystal structures of vinculin (PDB codes 1ST6 and 1TR2) and α‐catenin (4IGG), both of them peripheral proteins involved in interactions with integral membrane proteins and/or with membrane lipids (Pokutta *et al*., 2008; Izard and Brown, 2016). Their functional similarities with the likely P3 roles and activities are obvious.

### 2.4 A conserved amino acid residue in the P3 C‐terminal region is the main determinant for flower stalk elongation

**FIGURE 5:**
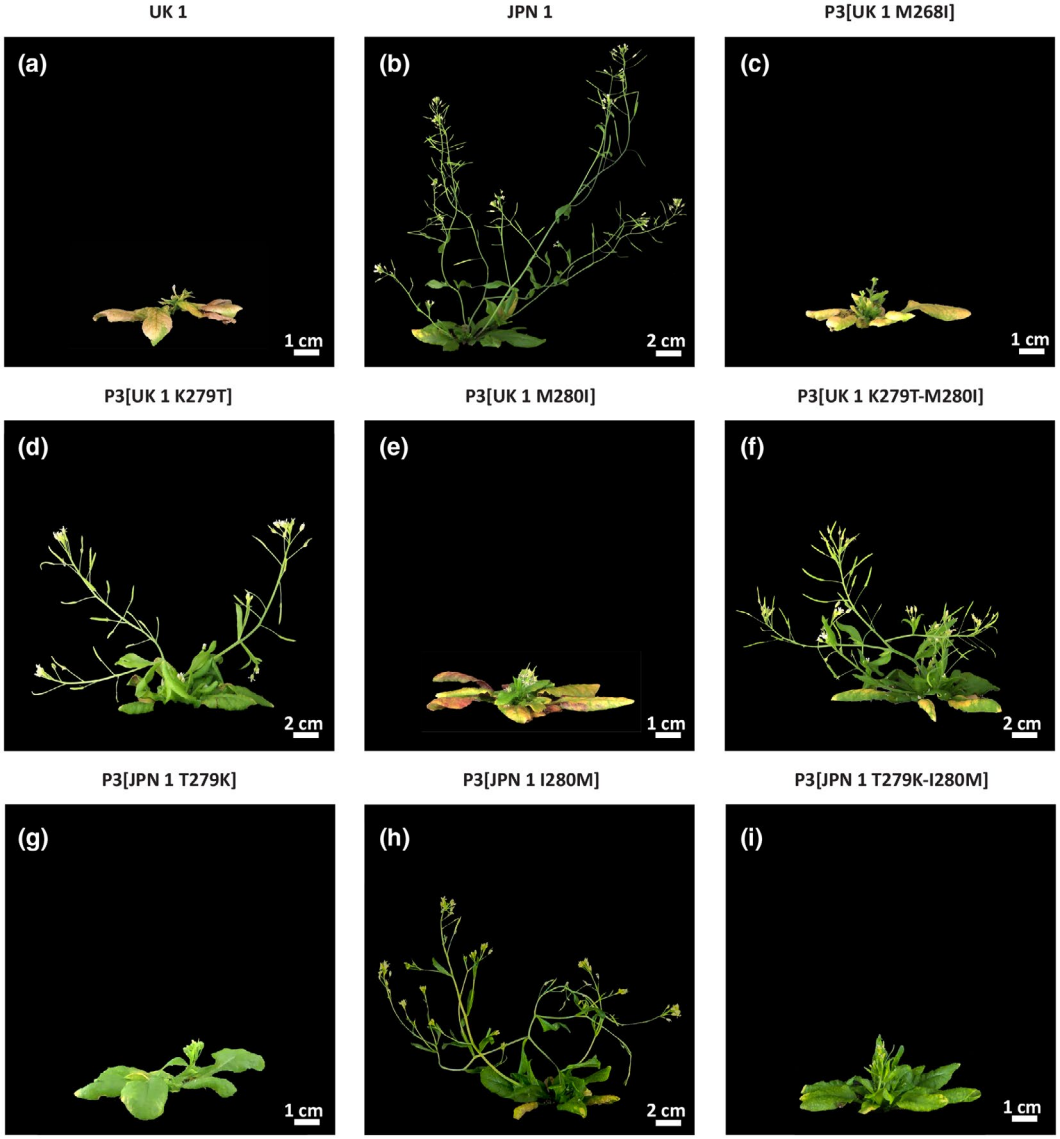
P3 single‐point mutation effects on *Arabidopsis* flower stalk elongation. (a) UK 1‐infection; (b) JPN 1‐infection; (c)–(f) mutants in positions 268 or 280 of P3[UK 1] do not change phenotypes, but lysine in position 279 swapped phenotypes; (g)–(i) converse changes in JPN 1. Pictures were taken 15 days postinoculation

To finely map the stalk elongation determinant within the P3 C‐terminal region (subdomain in the model), we tested some additional sequenced TuMV isolates to classify them as stalk‐allowing or ‐arresting. Ten isolates were individually inoculated in *Arabidopsis* and stalk development was assessed. Five isolates allowed elongation and five did not. Isolate identification and alignments of the stalk‐relevant region are shown in Figure S6. Only three positions (268, 279, and 280; locations indicated in Figure 4) correlated perfectly between stalk development and a specific amino acid combination. All isolates arresting stalk development had the combination M_268_, K_279_, M_280_; the nonarresting ones were I_268_, T_279_, I_280_. We generated single amino acid mutants in the infectious clones (Sánchez *et al*., 1998; López‐González *et al*., 2017). All three single mutants were made for UK 1, and positions 279 and 280 in JPN 1. Double mutants were also made at positions 279–280. Following inoculation, stalk elongation was assessed at 15 dpi (Figure 5), a time at which no significant differences in viral titres between the two strains were found previously (Manacorda *et al*., 2013). Of the single mutants, only those in position 279 (UK 1 K279T; JPN 1 T279K) were able to interchange the stalk growth pattern (Figure 5d,g). Mutant M268I allowed a very early elongation that completely halted by approximately 10 dpi (Figure 5c). JPN 1 double mutant 279–280 also changed the phenotype (Figure 5i). Thus, from the mutant analysis, the amino acid at position 279 was identified as the main determinant of stalk elongation.

### 2.5 The P3 amino acid determining flower stalk growth also determines P3 intracellular movement and accumulation pattern

**FIGURE 6:**
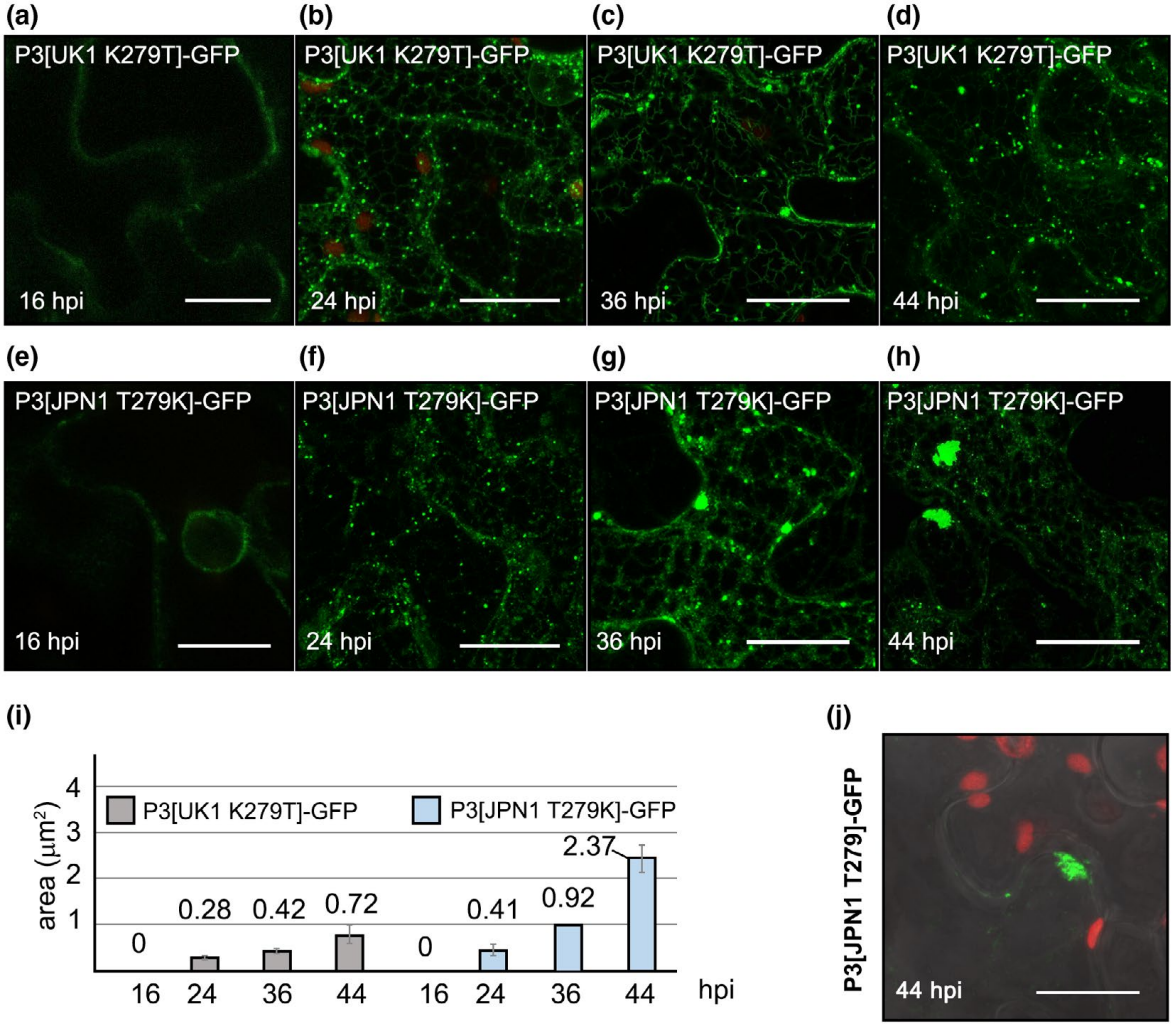
Time course of subcellular localization of *Nicotiana benthamiana* transiently expressed mutant TuMV P3‐GFPs. A single P3 amino acid change affects accumulation pattern: (a)–(d) threonine in P3[UK 1] position 279 reduced big aggregate formation; (e)–(h) and (j) lysine in P3[JPN 1] position 279 led to fluorescence accumulation close to cell periphery; (i) average sizes of punctate structures at different times; (j) *z*‐series stack projection showing a large aggregate of P3[JPN1 T279K]‐GFP near the cell periphery at 44 hr postinoculation (hpi). Except for (a) and (e) (single‐scan images), the remaining images are *z*‐series stack projections. Scale bars are 20 µm

The mutant and wild‐type P3 proteins fused to GFP were transiently expressed in *N. benthamiana* to investigate their accumulation and localization patterns over time (16, 24, 36, and 44 hpi). The progression of fluorescence distribution was swapped in the mutants (Figure 6). Figure 6a–d shows how P3[UK1 K279T]‐GFP slowed down accumulation in large aggregates, which only reached an average of 0.72 µm^2^, not significantly different from that obtained for P3[JPN1]‐GFP at the same time (0.53 µm^2^, Figure 6i, *q* = 1.86, *p* = .97), but considerably smaller than that observed for P3[UK1]‐GFP (5.41 µm^2^, Figure 1i, *q* = 39.58, *p* < .0001). Moreover, P3[UK1 K279T]‐GFP maintained a large portion of the fluorescence still labelling the cortical ER at 44 hpi (ER:punctate structures fluorescence ratio 5.1 ± 1.8), a behaviour more typical of the wild‐type P3[JPN1]‐GFP. In contrast, P3[JPN1 T279K]‐GFP progressively induced accumulation of large aggregates (Figure 6e–h) compared to P3[JPN1]‐GFP at 36 hpi (average 0.92 vs. 0.41 µm^2^, Figures 1i and 6i, *q* = 22.53, *p* = .033) and 44 hpi (average 2.37 vs. 0.53 µm^2^, Figures 1i and 6i, *q* = 20.8, *p* < .0001). However, P3[JPN1 T279K]‐GFP aggregates never reached the average size of those induced by P3[UK1]‐GFP at 36 hpi (average 0.92 vs. 2.53 µm^2^, Figures 1i and 6i, *q* = 13.56, *p* < .0001) or 44 hpi (average 2.37 vs. 5.41 µm^2^, Figures 1i and 6i, *q* = 28.10, *p* < .0001). Two‐way ANOVA also showed statistical significance between P3 mutant variants (*F* = 202.8, *p* < .0001), time (*F* = 189.4, *p* < .0001), and the interaction between P3 mutant variants and time (*F* = 76.9, *p* < .0001), indicating that differences in size between P3[UK1 K279T]‐GFP and P3[JPN1 T279K]‐GFP aggregates increased over time as described for wild‐type proteins. However, P3[JPN1 T279K]‐GFP cytoplasmic fluorescence fading was only occasionally observed (data not shown). Like P3[UK1]‐GFP aggregates, those induced by P3[JPN1 T279K]‐GFP were most likely in close association with the cell periphery, because a dark area between adjacent cells can be observed, probably the middle lamella (Figure 6j, and 360° 3D Video S10). Videos S11 and S12 show P3[UK1 K279T]‐GFP punctate structures movement at 2 dpi, and of P3[JPN1 T279K]‐GFP static aggregates with some mobile punctate structures at 2 dpi, respectively.

The results obtained allow a tight association to be established between the amino acid present at position 279 of P3 (K or T), the intracellular patterns of P3 movement and accumulation of the protein, and the arrest of the growth or the elongation of TuMV‐infected *Arabidopsis* flower stalks.

### 2.6 Mutants altering flower stalk elongation and P3 movement/accumulation patterns can be assigned to local changes of P3 surface solvent‐exposition and electrostatic potential

**FIGURE 7:**
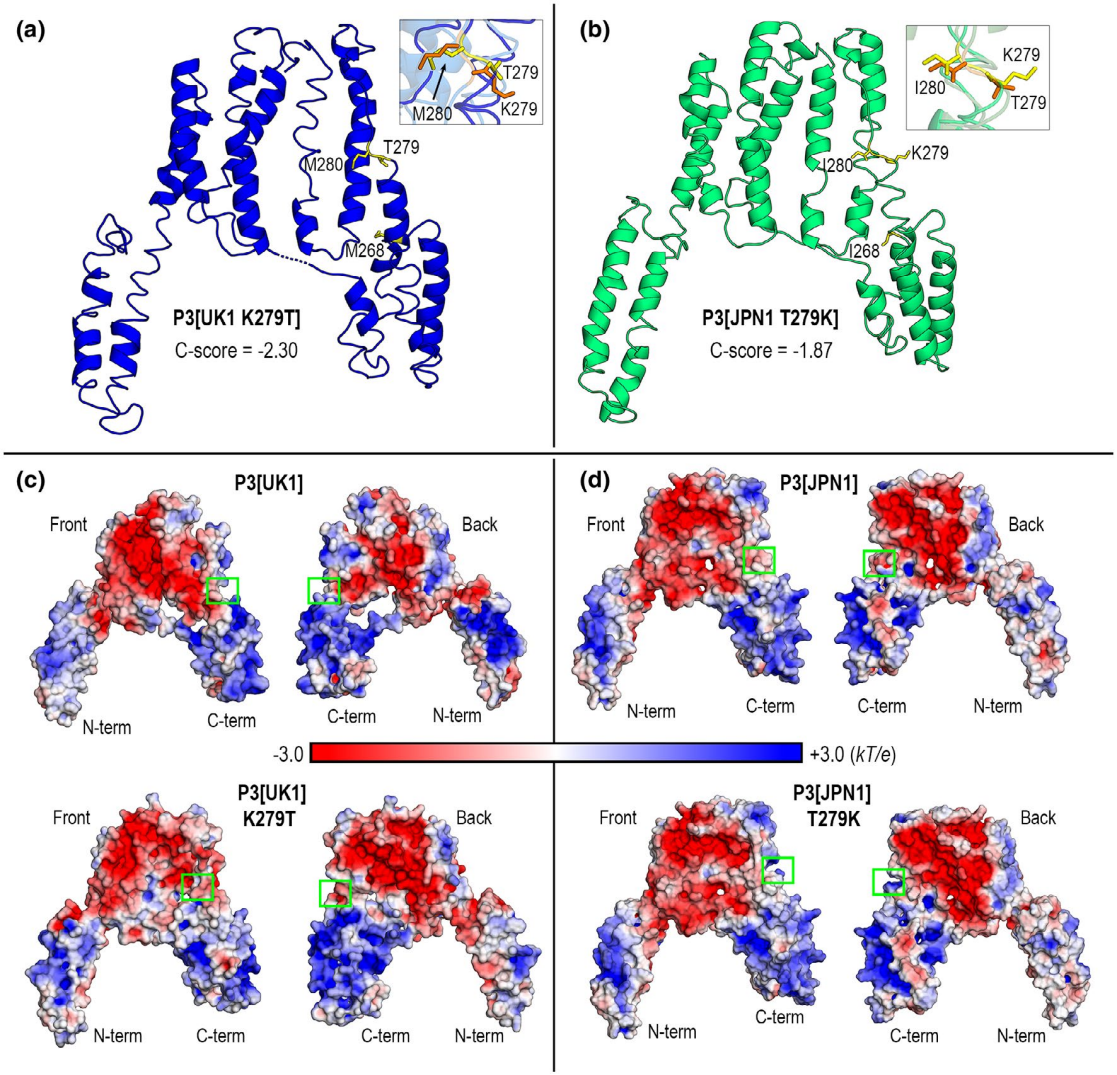
In silico modelled structures of P3‐279 mutants. Ribbon diagrams of I‐TASSER predicted structures of (a) P3[UK1 K279T] and (b) P3[JPN 1 T279K]. Side chain conformations of amino acids 279 and 280 are shown in the insets as sticks in orange and yellow corresponding to wild‐type and mutant forms, respectively. (c) Poisson–Boltzmann (PB) electrostatic potentials (EPs) mapped onto P3 mutants surface. Front views correspond to orientations shown in (a) and (b); back views are obtained upon a 180° rotation around a vertical axis. Surface patches with electrostatic changes arising from mutations at position 279 are enclosed in green boxes. PB‐EP values are given in *kT*/*e* units, *k* being Boltzmann's constant, *T* absolute temperature (298 K) and *e* electron charge

Elongation‐altering postion 279 mutants were independently modelled with I‐TASSER as done earlier for wild‐type forms. Single position 279 mutants slightly modify local backbone geometry (Figure 7). However, a greater change is seen in the orientation of side chains, more protuberant and exposed to the solvent when residue 279 is lysine (Figure 7), something all the more reasonable considering that lysine is positively charged and has a longer chain than threonine. Therefore, from a purely structural standpoint the effect on stalk elongation and movement/accumulation of P3 can be associated with a larger exposition of lysine to the aqueous environment.

However, in the study of protein–membrane interactions electrostatic potentials weigh more than local structural changes, or even protein global shapes (Davis and McCammon, 1990; Honig and Nicholls, 1995). So, we computed electrostatic potentials for both P3 proteins and their mutants by solving the nonlinear Poisson–Boltzmann (PB) equation with the program APBS (Davis and McCammon, 1990; Baker *et al*., 2001). The results are shown in Figure 7c,d. The front and back views of the protein surfaces reveal a marked electrostatic polarity between the dominantly negative core domain and the two dominantly positive subdomains in both wild type and mutants. Predominance of surface regions with positive electrostatic potential is a well‐known feature of membrane‐interacting proteins (e.g., Mulgrew‐Nesbitt *et al*., 2006). As for the position 279 mutants, the electrostatic effect is noticed as an increase of the positive electrostatic nature in a surface patch, larger than the strictly associated side chain, when the amino acid 279 is lysine instead of threonine (Figure 7c). Because T279 in JPN 1 or in mutated UK 1 slows down accumulation in larger aggregates and allows stalk elongation, whereas K279 has the opposite effects, one might correlate these traits to electrostatic features. Note also that position 279 lies at the external side of a structural region joining the core domain and the C‐terminal subdomain in both P3s (Figures 4 and 7a,b).

### 2.7 Transiently expressed fluorescent TuMV P3 co‐expressed with fluorescent 6K2

**FIGURE 8:**
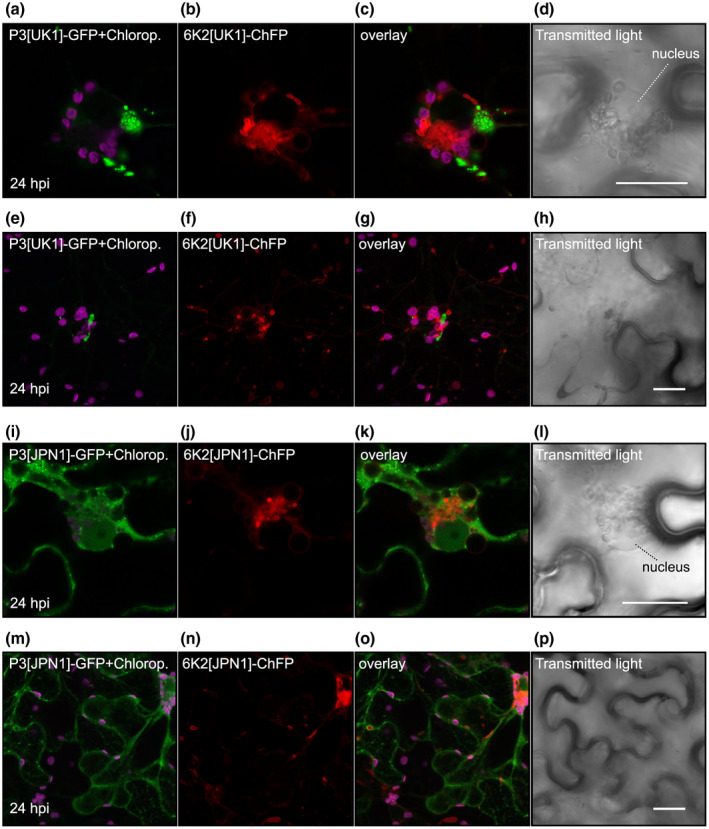
Subcellular localization of TuMV P3‐GFP and TuMV 6K2‐ChFP after transient expression in *Nicotiana benthamiana* epidermal cells. Purple fluorescence is due to chlorophyll autofluorescence. (a)–(h) Distribution pattern of P3[UK1]‐GFP, 6K2[UK1]‐ChFP, and chloroplast fluorescence. A detail of the perinuclear region is shown in (a)–(d) and a general view of the cell in panels (e)–(h). (i)–(p) Similar to above, but with P3[JPN1]‐GFP and 6K2[JPN1]‐ChFP. Images correspond to *z*‐series stack projections. Scale bars are 20 µm

P3 has been reported to co‐localize with the 6K2 protein, the main one responsible for the extensive endomembrane rearrangement and chloroplast association in potyvirus infections, even when expressed alone (Wei and Wang, 2008; Cotton *et al*., 2009; Grangeon *et al*., 2012; Cui *et al*., 2017). To address the co‐localization issue of P3 and 6K2 in UK 1 and JPN 1, *N. benthamiana* leaves were agroinfiltrated with constructs expressing fusion proteins 6K2[UK1]‐ChFP or 6K2[JPN1]‐ChFP. The results in Figure S7 show that both 6K2 proteins indeed co‐localized with the chloroplast envelope and the stroma (purple‐coloured chlorophyll autofluorescence). The same constructs were co‐agroinfiltrated with the P3‐GFP fusion proteins of their corresponding isolates (Figure 8). In these experiments GFP‐P3 fusion proteins were also included to discard the possibility of artefacts due to the presence of GFP in the critical C‐ter P3 region, with similar results (not shown). At 1 dpi, co‐expression of P3[UK1]‐GFP together with 6K2[UK1]‐ChFP revealed the joint perinuclear presence of both proteins and chloroplasts (Figure 8a–d shows a detail of the perinuclear region and Figure 8e–h shows a general view of the cell). This joint accumulation of the viral proteins appears to be induced by 6K2 because normal distribution of P3 co‐expressed together with free ChFP was not altered (not shown). In this sense, aggregates of chloroplast, P3[UK1]‐GFP, and 6K2[UK1]‐ChFP moved thorough the cytoplasm (Video S13) until they reached the perinuclear region (Video S14). A different result was obtained for P3[JPN1]‐GFP. At 1 dpi, co‐expression of 6K2[JPN1]‐ChFP did not alter P3 distribution. 6K2[JPN1]‐ChFP continued to accumulate perinuclearly together with a large amount of chloroplasts, but P3 was found in the form of small mobile particles across the cytoplasm, moving independently of 6K2[JPN1]‐ChFP (Figure 8i–p, Videos S15 and S16). 6K2 forms chloroplast‐interacting ER‐derived vesicles (Wei *et al*., 2010, 2013; Grangeon *et al*., 2012; Cui *et al*., 2017), and UK 1 P3 is present in these membranous structures, as described for TEV and TuMV by other authors (Cui *et al*., 2010, 2017; Chai *et al*., 2020), but the behaviour of JPN 1 P3 in relation to its 6K2 partner has not been previously described.

### 2.8 Transiently expressed fluorescent TuMV P3 in the context of TuMV infections

The differential behaviour of the P3 proteins when co‐expressed with their corresponding 6K2 proteins prompted us to wonder if their expression would also be differentially affected in the context of a viral infection. Constructs expressing P3[UK1]‐GFP, P3[JPN1]‐GFP, GFP‐P3[UK1], and GFP‐P3[JPN1] were agroinfiltrated in symptomatic leaves of infected *N. benthamiana* plants previously infected with their corresponding viral strains as infectious clones (Figure S8d,j). Agroinfiltrations in leaves from healthy plants were used as controls (Figure S8a,g). Because symptoms appearance varies between both TuMV isolates, constructs were agroinfiltrated 4 days after TuMV‐UK1 inoculation and 10 days after TuMV‐JPN1 inoculation. Confocal microscopy observations were done at 36 hpi. As expected, P3[UK1]‐GFP and GFP‐P3[UK1] accumulated in the ER and in large peripheral and cytoplasmic aggregates (Figure S8b,c). A similar fluorescence distribution was observed in infected tissue, except that P3[UK1]‐GFP and GFP‐P3[UK1] also accumulated around the nuclei (Figure S8e,f). No differences in the fluorescence distribution patterns of P3[JPN1]‐GFP and GFP‐P3[JPN1] were found between healthy and infected tissue (Figure S8h,i vs. Figure S8k,l, respectively). In all of them, P3[JPN1] fusion proteins labelled small punctate structures broadly distributed throughout the cytoplasm.

To ensure that all observations were actually made in infected cells, we also agroinfiltrated the constructs in symptomatic leaves of *N. benthamiana* previously inoculated with TuMV‐UK1 and TuMV‐JPN1 GFP‐tagged infectious vectors (Figure S9), which allowed the identification of infected cells. No differences were observed with respect to the results described above regarding the distribution in healthy or infected plants. Taken together these results indicate that the differences in behaviour observed between P3[UK1] and P3[JPN1] when expressed in healthy tissue parallels the results obtained in the context of a TuMV infection.

## 3 DISCUSSION

P3 was identified as the main mediator of flower stalk elongation or arrest in TuMV‐infected *Arabidopsis* plants. Its C‐terminal domain contains the determinant of this developmental trait (Sánchez *et al*., 2015). Recent work (Cui *et al*., 2017) has shown the implication of the protein in viral intercellular movement, virus replication, co‐localization with the other viral membrane protein (6K2), and formation of perinuclear chloroplast‐bound 6K2 vesicles. All these P3‐mediated processes are influenced by determinants in the P3 C‐terminal region. We also recently identified a molecular motif within this region as determinant of the apparent nonhost resistance of Ethiopian mustard to the JPN 1 isolate (Sardaru *et al*., 2018). All these findings provided an adequate context for P3 comparisons of two TuMV isolates (UK1 and JPN 1) having differential effects on *Arabidopsis* development.

When P3‐GFP fusion proteins were transiently expressed in *N. benthamiana*, they associated with the ER, highlighting the reticulate but also moving along and forming cytoplasmic puncta of aggregated protein. Both fusion proteins associated peripherally with the ER. An association of the P3‐GFP fusion proteins with the Golgi apparatus was not found. Both fusion proteins formed large fluorescent aggregates, but at different rates. UK 1 P3 already formed large aggregates 1 day after inoculation, whereas it took one more day for the JPN 1 isolate to do so. In both cases most aggregates formed close to the cell periphery. In our view, this behaviour of the P3 protein is related to its proposed role as a protein involved in the viral intercellular movement. That the protein alone is able to move by ER streaming towards the cell periphery and aggregate at certain locations close to the periphery speaks of a probable role in transporting viral components. In this regard, the recent proposal of a role for specific ER subdomains, such as cortical microtubule‐associated ER sites (cMERs) or ER–plasma membrane contact sites, as hubs for the control of viral intercellular movement through plasmodesmata (Pitzalis and Heinlein, 2017), would fit nicely with the behaviour of TuMV P3, which would move towards these structures, aggregating in them. An important difference between the P3 proteins of both isolates would be the rate of doing so. We previously reported a slightly higher level of virus accumulation in UK 1‐infected *Arabidopsis* plants early infections, in comparison with JPN 1‐infected (Manacorda *et al*., 2013), probably related to the intracellular movement rate of each virus. Both P3 proteins differ in the presence/absence of a lysine residue. Remarkably, a lysine residue has been shown as critical in the ER lateral translocation of a viral movement protein (Serra‐Soriano *et al*., 2014).

Association of peripheral proteins with membranes is looser than integral proteins and may involve lipids and/or other membrane proteins (Monje‐Galván and Klauda, 2016). The 3D structure of peripheral proteins must govern the type of association with their membrane counterparts, but the TuMV P3 3D structure has not been resolved. In the absence of this information, refined modelling should shed light on the differential ER‐association of both P3 proteins. We relied on I‐TASSER for modelling, the top prediction method available. Secondary structures were identified with DSSP. The outcome of these methods did not contain predictions of TMDs. Rather the model consists of two large domains, a core helical bundle, and two separated helical subdomains, N‐terminal and C‐terminal regions. The N‐terminal subdomain was quite similar for both isolates, but the helical arrangement of the C‐terminal was spatially different, and the spatial position of the C‐terminal residue was very different.

A further refinement of the stalk elongation/arrest determinant within the C‐terminal region was sought. For this we generated point mutations at positions highly conserved within the C‐terminal region of TuMV isolates that allow or arrest stalk elongation. Sequence comparisons identified three candidate positions for mutagenesis. Single mutations were performed at these positions in the TuMV infectious clones, interchanging their amino acids. Inoculation of the mutants in *Arabidopsis* identified P3 position 279 as the determinant for the trait. The results prompted us to test the subcellular behaviour of the mutant proteins, which were also interchanged, confirming the association between both characteristics.

3D models of the mutants offered a plausible reason for the association found, linking the protein subcellular behaviour to a marked change in the surface electrostatic potential. P3‐ER association could be affected by this alteration in the electrostatic potential of the region, most likely the region interacting with some integral membrane component. At present the nature of this potential interactor is unknown, although in this context it is relevant to consider the recent finding of the direct interaction of *Soybean mosaic virus* (another potyvirus) P3 with the reticulon homology domain protein of soybean (Cui *et al*., 2018), an integral ER membrane protein.

A final relevant difference was found in the subcellular behaviour of the two P3 proteins when transiently expressed together with another potyviral membrane protein, 6K2. For UK 1 P3 we confirmed the behaviour found previously for TuMV P3, a co‐localization of both membrane proteins in association with ER‐derived 6K2‐induced vesicles and chloroplasts in the close periphery of the nucleus, in addition to its normal ER reticulate location. In this case, P3 would be “dragged” by 6K2 to co‐localize, although interestingly no direct interaction has been so far found between the two proteins. The presence of P3 in the 6K2‐induced membranous structures has been related to a critical role of P3 in the formation and functioning of the VRC (Cui *et al*., 2017). In the case of JPN 1 P3, the protein was not found co‐localizing with perinuclear 6K2 vesicles. Interestingly, the P3 domain mediating the interaction with the vesicles is the C‐terminal domain, which also contains the determinant of stalk elongation and subcellular movement dynamics of the protein. This result opens up the possibility of alternative ways through which P3 would move 6K2‐containing VRCs along the ER, but this aspect should be further investigated.

Interestingly, when the fluorescent P3 proteins were transiently expressed in cells infected with their corresponding TuMV strain, their localizations and dynamics were highly similar to those found when co‐expressed with their corresponding 6K2 proteins, thus highlighting both the differential behaviours of the two P3 proteins within cells and the relevance of 6K2 in relation to P3 in the infection process.

Taking together the findings of this comparative study, a model based on them can be proposed for the role of P3 in TuMV infections, although still an incomplete one. This viral membrane protein peripherally associated with the ER membrane, possibly through a direct interaction with some ER integral protein, would undergo a process of cytoplasmic streaming. This would be an actomyosin‐dependent process leading the protein towards specific locations close to the cell periphery. Most likely this process is a main mediator of the intracellular movement of VRCs on their way towards plasmodesmata. This model implies that the ER‐mobile P3 must interact with the 6K2‐containing VRCs, the viral complex spreading the infection to the neighbour cells (Movahed *et al*., 2017), which would be the cargo towards the cell periphery. The specifically dedicated intercellular movement viral proteins (CI and P3N‐PIPO) would then play their role as mediators of the movement through plasmodesmata. P3 may or may not play a role in the formation of the VRC, but it would be its carrier, although at different rates depending on viral strains. Very recently a model for the role of TuMV P3 protein in the viral cell‐to‐cell movement has been proposed (Chai *et al*., 2020). Although not specifically mentioned in the publication, the TuMV isolate on which this model is based is most probably of the UK 1 type. The main novelty of this model is that it shows a direct interaction of P3 and P3N‐PIPO via the shared N‐terminal domain of both proteins, an interaction that would allow the connection between CI inclusions and the 6K2‐containing vesicles to be established. We discarded previously a possible role of P3N‐PIPO as a determinant of flower stalk elongation (Sánchez *et al*., 2015), so we did not study this movement‐dedicated fusion protein in our work. However, the proposed model is fully compatible with the one described in this paper. P3 would interact with the 6K2 vesicles forming a complex also containing P3N‐PIPO, although the major localization of P3N‐PIPO to the cell surface suggests that the main incorporation of P3N‐PIPO into the vesicles would occur after the P3‐mediated ER streaming of the vesicles. The presence of some P3N‐PIPO in the ER‐moving vesicles is also possible, but this would not affect our main proposal of a differential moving rate between both isolates.

Finally, the link of the subcellular behaviour of the protein and its 3D structure with the alterations in the developmental phenotype of the infected plant is still not fully resolved. The phenotypes found for each isolate (flower stalk arrest in UK 1 and creeping growing habit in JPN 1) point to different types of cell wall alterations depending on virus strain. Work in progress is currently devoted to the characterization of these alterations.

## 4 EXPERIMENTAL PROCEDURES

### 4.1 Construction of binary vectors for fluorescent‐tagged P3 recombinant proteins

Gene sequences were amplified by PCR. The resulting fragments were purified and digested with *Nhe*I and *Nco*I. Digested fragments were cloned between the CaMV 35S promoter and the PoPit terminator into modified pBSIIKS(+), which allows cloning PCR products to directly generate GFP and ChFP fusion proteins. The resulting clones, described in detail in the Results section, were digested with *Hin*dIII and cloned into pMOG800 binary vector (Knoester *et al*., 1998). In the case of 6K2 fusion proteins, *Xho*I/*Eco*RI or *Hin*dIII enzymes were used to liberate the cassette from pBSIIKS (+). At the same time, pMOG800 vector was digested by the same enzymes. The primer sequences used for cloning are listed in Table S1.

### 4.2 Fluorescent organelle markers

The fluorescent organelle markers used were rat α–2,6‐sialyltransferase transmembrane domain fused to ChFP (STtmd–ChFP) as Golgi marker, a form of ChFP(mCherry)/GFP containing the N‐terminal signal peptide from an *A. thaliana* vacuolar basic chitinase and the ER retrieval sequence KDEL fused at the 3′ end (ChFP/GFP–KDEL) as ER marker, talin protein fused to dsRFP (dsRFP‐Talin) as actin microfilament marker, and α‐tubulin fused to ChFP (αTubulin‐ChFP) as microtubule marker (Genovés *et al*., 2010).

### 4.3 Protein expression in *N. benthamiana*

Binary constructs were electroporated into *Agrobacterium tumefaciens* C58C1. Transformed bacteria were grown overnight in Luria Bertani medium with antibiotics (50 μg/ml kanamycin, 100 μg/ml rifampicin). Cultures were centrifuged and resuspended in 10 mM MgCl_2_, 10 mM MES pH 5.6, with 150 μM acetosyringone. Bacterial optical density at 600 nm was adjusted to 0.2. Four‐week‐old *N. benthamiana* plant leaves were infiltrated with the bacteria and kept in growth chamber under 16 hr light at 25 °C and 8 hr dark at 22 °C.

### 4.4 Confocal laser scanning microscopy

Subcellular localization of fluorescent proteins was performed with an inverted Zeiss LSM 780 confocal microscope. eGFP and ChFP/dsRFP fluorescence was visualized by 488 and 561 nm laser excitation, respectively. The emission detection windows were 492–532 and 590–630 nm, respectively. Chlorophyll excitation wavelength was 488 nm, fluorescence was detected above 700 nm.

### 4.5 Microsomal fractionation and immunoblotting

Five grams of *N. benthamiana* leaves individually expressing P3‐GFP fusion proteins were homogenized in 10 ml of lysis buffer (20 mM HEPES, pH 6.8; 150 mM potassium acetate; 250 mM mannitol, 1 mM MgCl_2_). Plant leaves transiently expressing GFP‐p7B were used as a source of transmembrane protein of the ER and Golgi apparatus (Genovés *et al*., 2010). Large cellular debris was removed by centrifugation at 8,000 × g for 10 min, and the supernatant was Miracloth‐filtered. The total protein fraction was aliquoted in five aliquots, which were ultracentrifuged at 100,000 × g for 30 min to generate the soluble (S100) and microsomal (P100) fraction. The S100s were stored and the P100s resuspended in 2 ml of lysis buffer supplemented with 0.1 M Na_2_CO_3_, pH 11.5, 4 M urea, 8 M urea, or 0.1% Triton X‐100. Treatments were performed on ice for 30 min. After incubation, aliquots were ultracentrifuged at 100,000 × g for 30 min and pellets resuspended in 400 µl of lysis buffer. Samples from each fraction were analysed by 12% SDS‐polyacrylamide electrophoresis (PAGE), and subsequently transferred to polyvinylidene difluoride membranes for immunoblotting with a polyclonal antibody to Nt‐GFP.

### 4.6 Point mutations in TuMV infectious clones

Mutations were introduced in P3 by overlap extension PCR cloning. To introduce each mutation, three PCRs were performed. The first two PCRs were done with an external primer (E1/E2) and internal primer (I1/I2) carrying the mutation(s) to be introduced. The products of these PCRs were mixed and used as templates for the third PCR, with the E1 and E2 primers (Table S1). All PCRs were performed using *Pfu* DNA polymerase. PCR fragments were cloned into Zero Blunt Topo and sequenced in an external sequencing service (Secugen). Vectors carrying the mutations were digested with *Sna*BI and *Btg*I and cloned into the infectious clone of UK 1 (previously digested with *Sna*BI and *Btg*I) in position 3305–4011 of the clone (Sánchez *et al*., 1998). The same procedure was followed for JPN 1, but in this case we introduced the mutated fragment in position 2781–4167 using the restriction enzyme *Sal*I (López‐González *et al*., 2017). Stability of the mutations after *Arabidopsis* infection was confirmed by immunocapture reverse transcription PCR (IC‐RT‐PCR) (Nolasco *et al*., 1993) and sequencing. The monoclonal antibody used for IC‐RT‐PCR was Anti‐Poty and the primers are listed in Table S1.

### 4.7 Plant growth and virus inoculation

*Arabidopsis* plants were grown in controlled chambers and inoculated with crude sap from cDNA‐infected plants, as previously described (Sánchez *et al*., 1998; Lunello *et al*., 2007). Growth conditions were 21 °C (day) and 18 °C (night) in 16 hr/8 hr cycles. Inoculations were performed at stage 1.08 of *Arabidopsis* development (Boyes *et al*., 2001) and symptoms of infection started to become visible approximately 5 dpi. They were observed daily and recorded.

### 4.8 Latrunculin B treatments

A stock solution of Latrunculin B was prepared in dimethyl sulphoxide (DMSO) and diluted in water. For P3 movement experiments, *N. benthamiana* leaf discs transiently expressing P3‐GFP fusion proteins and dsRFP Talin (Genovés *et al*., 2010) were immersed into 1 μM LatB for 10 min. Controls were leaf discs from plants transiently expressing dsRFP‐talin and calreticulin sequence encoding KDEL at the 3′ end of GFP (GFP‐KDEL) (Genovés *et al*., 2010) immersed into DMSO. Tissue was visualized using an inverted Zeiss LSM 780 confocal microscope.

### 4.9 Image and statistical analysis

Image analysis was performed using FIJI software (US National Institutes of Health). Quantification of intracellular fluorescence was performed by manually drawing regions of interest (ROI). Particle area measure was performed using the analyse particles option. Projection of *z*‐series stack was done using the standard deviation method and 3D projection was done using the interpolation tool to eliminate the gaps for the final 3D outcome. Western blot intensity signal was measured on files from a Fujifilm LAS‐3000 Imager using Fuji Image Gauge v. 4.0 software. For the statistical analysis, two‐way ANOVA and Tukey's post hoc test at the 95% confidence level (α = .05) for pairwise comparison was applied to the data using GraphPad Prism v. 6.01 software.

### 4.10 3D Structures, protein surfaces, and electrostatic potentials

Model 3D structures were predicted with I‐TASSER (Roy *et al*., 2010; Yang *et al*., 2014). Secondary structure was identified with the DSSP program (Kabsch and Sander, 1983; Touw *et al*., 2015). Poisson–Boltzmann (PB) electrostatic potentials were computed solving the nonlinear PB equation with the Adaptive Poisson Boltzmann Solver APBS v. 1.4.1 program (Davis and McCammon, 1990; Baker *et al*., 2001). Sequential focusing multigrid calculations were performed with APBS at 3D grids of step size 0.5 Å at 298 K and 0.150 M salt concentration with dielectric constants 4 for proteins and 78.54 for water. The numerical output of PB potentials was saved in OpenDX scalar format and mapped onto molecular surfaces. Molecular graphics of structures, surfaces, and PB potentials were prepared and rendered with PyMOL v. 2.0.3 (The PyMOL Molecular Graphics System, Version 2.0 Schrödinger, LLC).

## Supporting information

Supplementary Material

Supplementary Material

Supplementary Material

Supplementary Material

Supplementary Material

Supplementary Material

Supplementary Material

Supplementary Material

Supplementary Material

Supplementary Material

Supplementary Material

Supplementary Material

Supplementary Material

Supplementary Material

Supplementary Material

Supplementary Material

Supplementary Material

Supplementary Material

Supplementary Material

Supplementary Material

Supplementary Material

Supplementary Material

Supplementary Material

Supplementary Material

Supplementary Material

Supplementary Material

## Data Availability

The data that support the findings of this study are available from the corresponding author upon reasonable request.
