# Supplementary figures and images for: Association between flower stalk elongation, an Arabidopsis developmental trait, and the subcellular location and movement dynamics of the nonstructural protein P3 of Turnip mosaic virus
Source: Mol Plant Pathol. 2020 Aug 1;21(10):1271–86. doi: 10.1111/mpp.12976 (PMC7488469; doi:10.1111/mpp.12976)

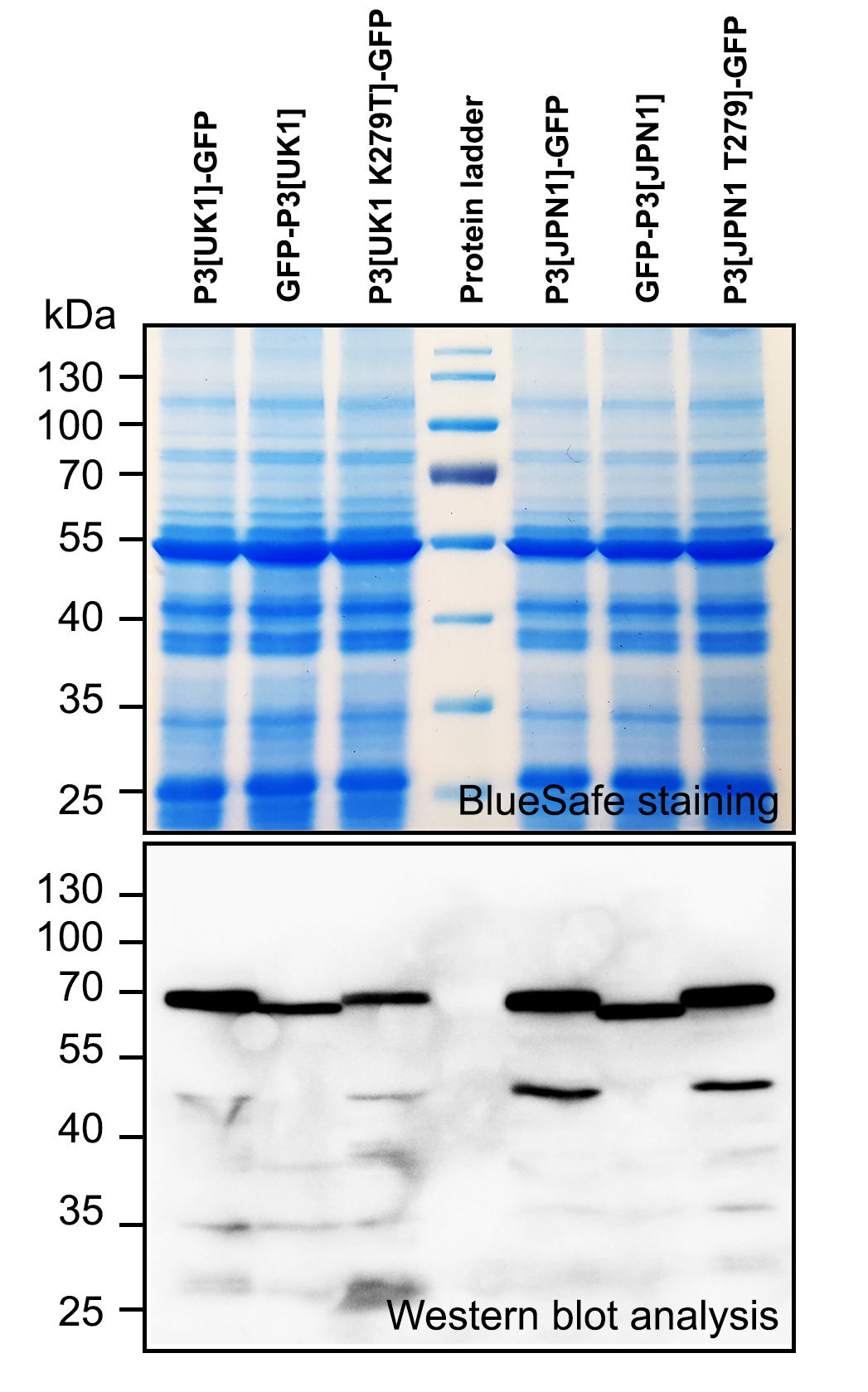

Supplement: Supplementary file 1 — Supplementary Material [file MPP-21-1271-s001.TIF]

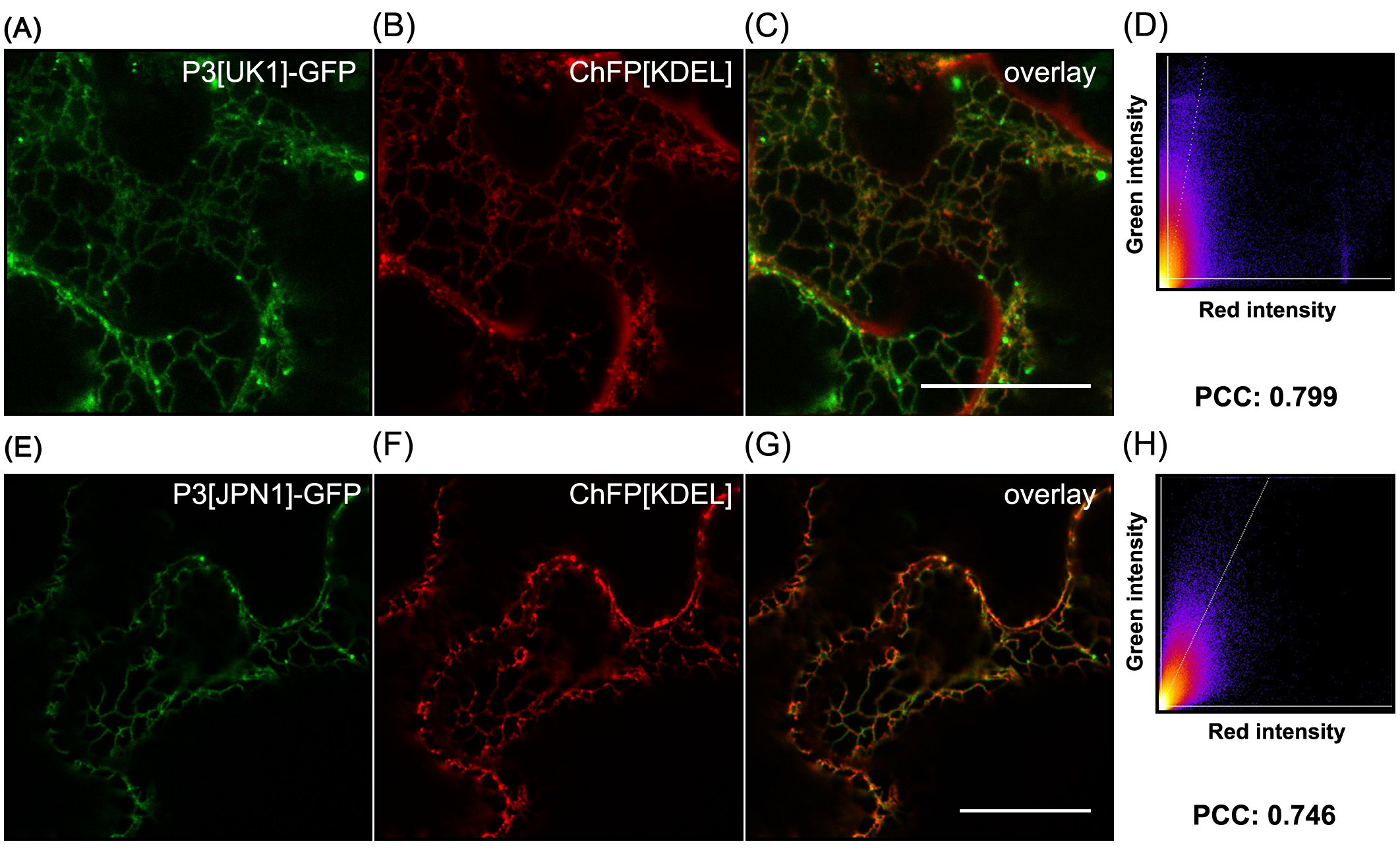

Supplement: Supplementary file 2 — Supplementary Material [file MPP-21-1271-s002.TIF]

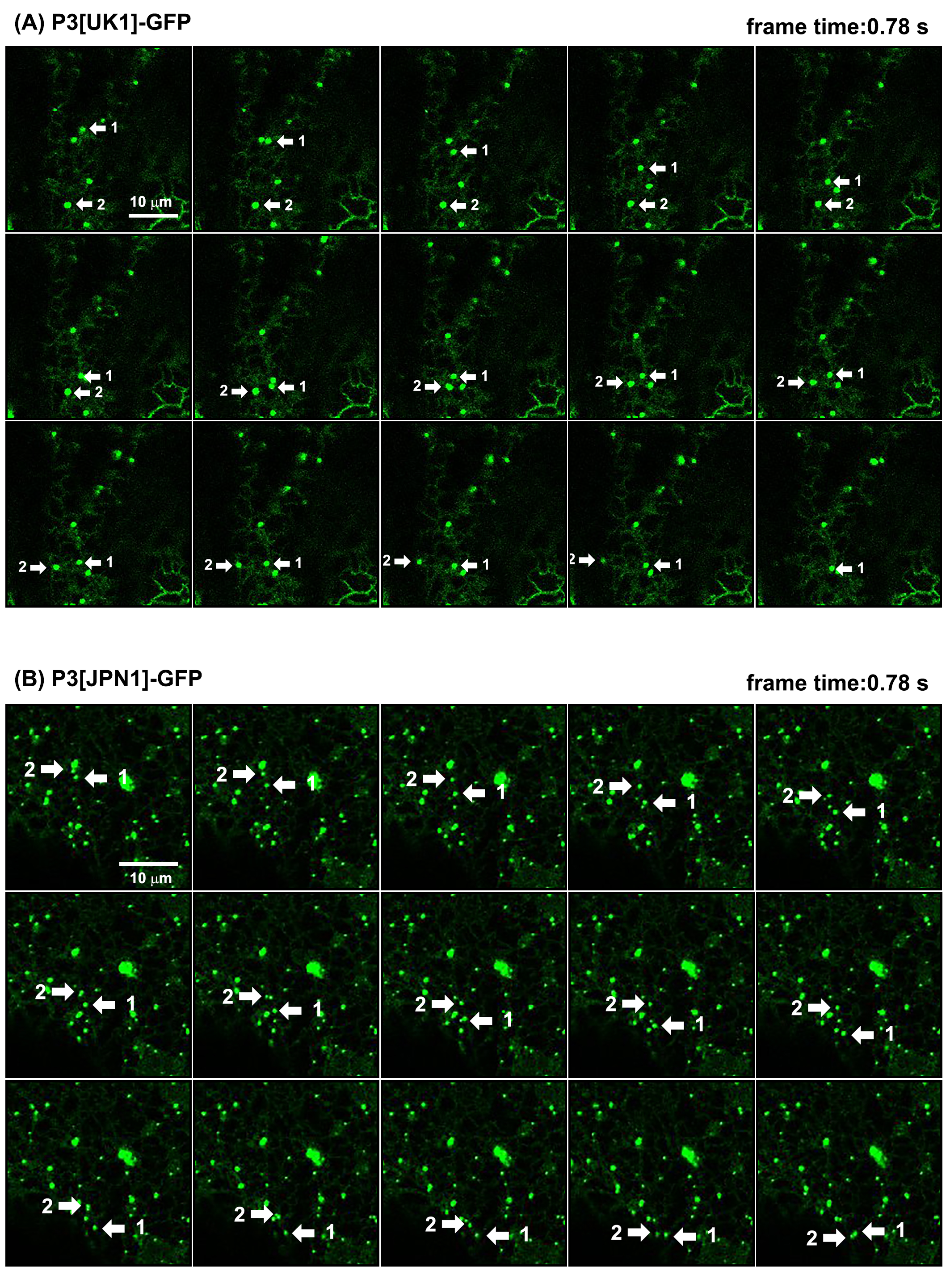

Supplement: Supplementary file 3 — Supplementary Material [file MPP-21-1271-s003.TIF]

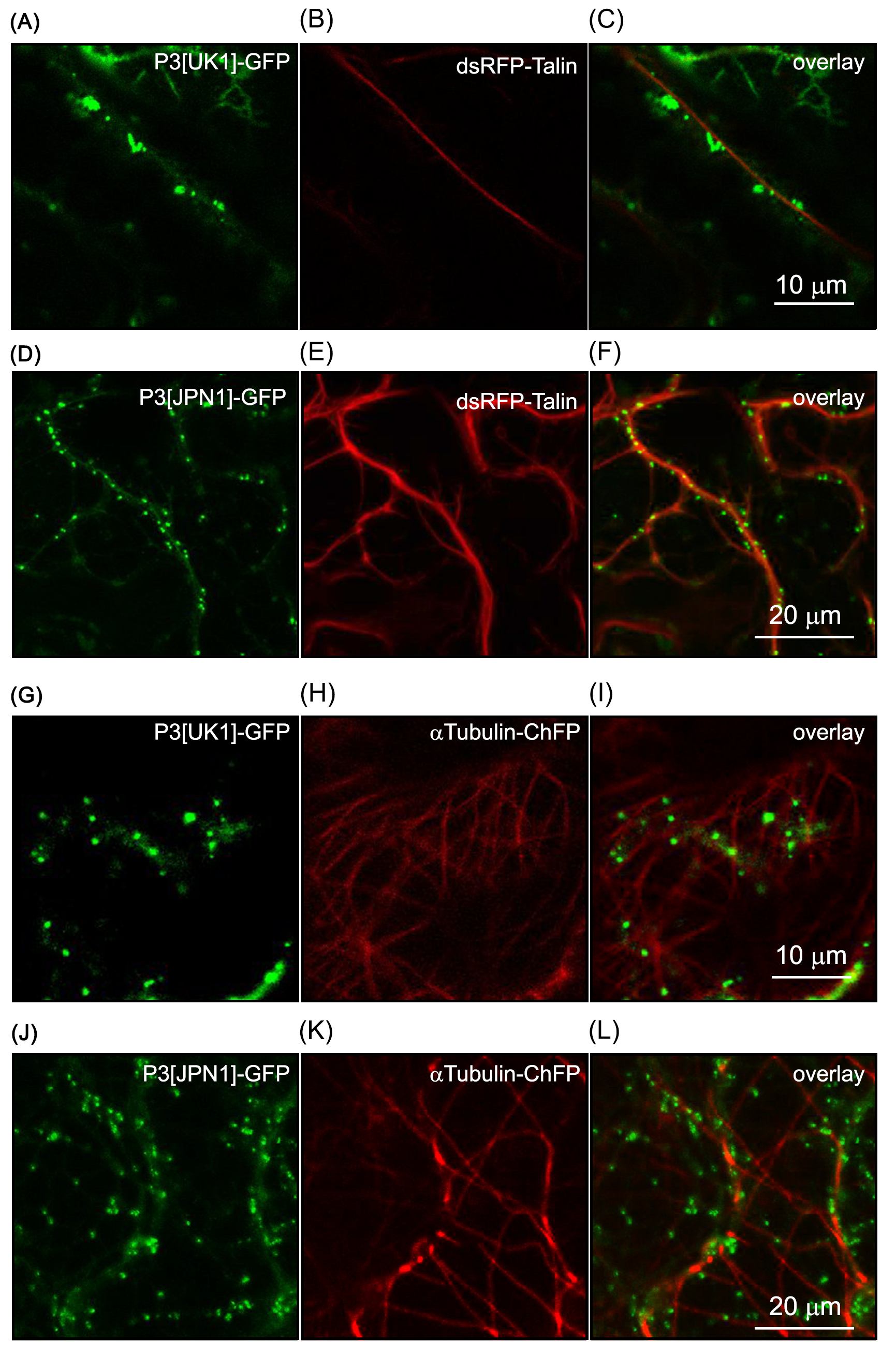

Supplement: Supplementary file 4 — Supplementary Material [file MPP-21-1271-s004.TIF]

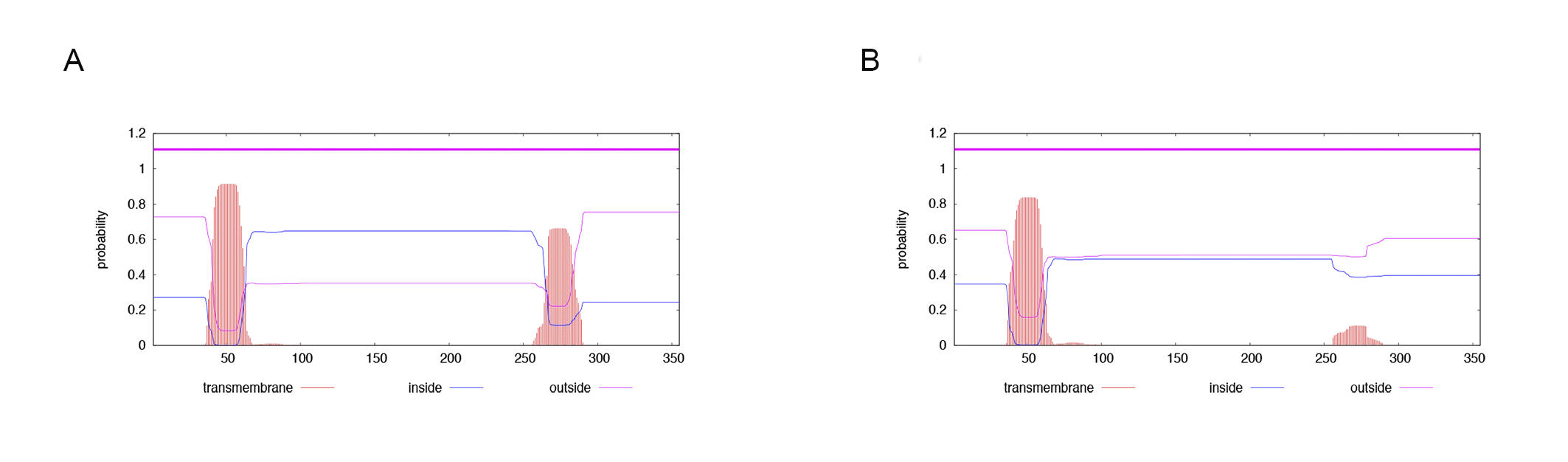

Supplement: Supplementary file 5 — Supplementary Material [file MPP-21-1271-s005.TIF]

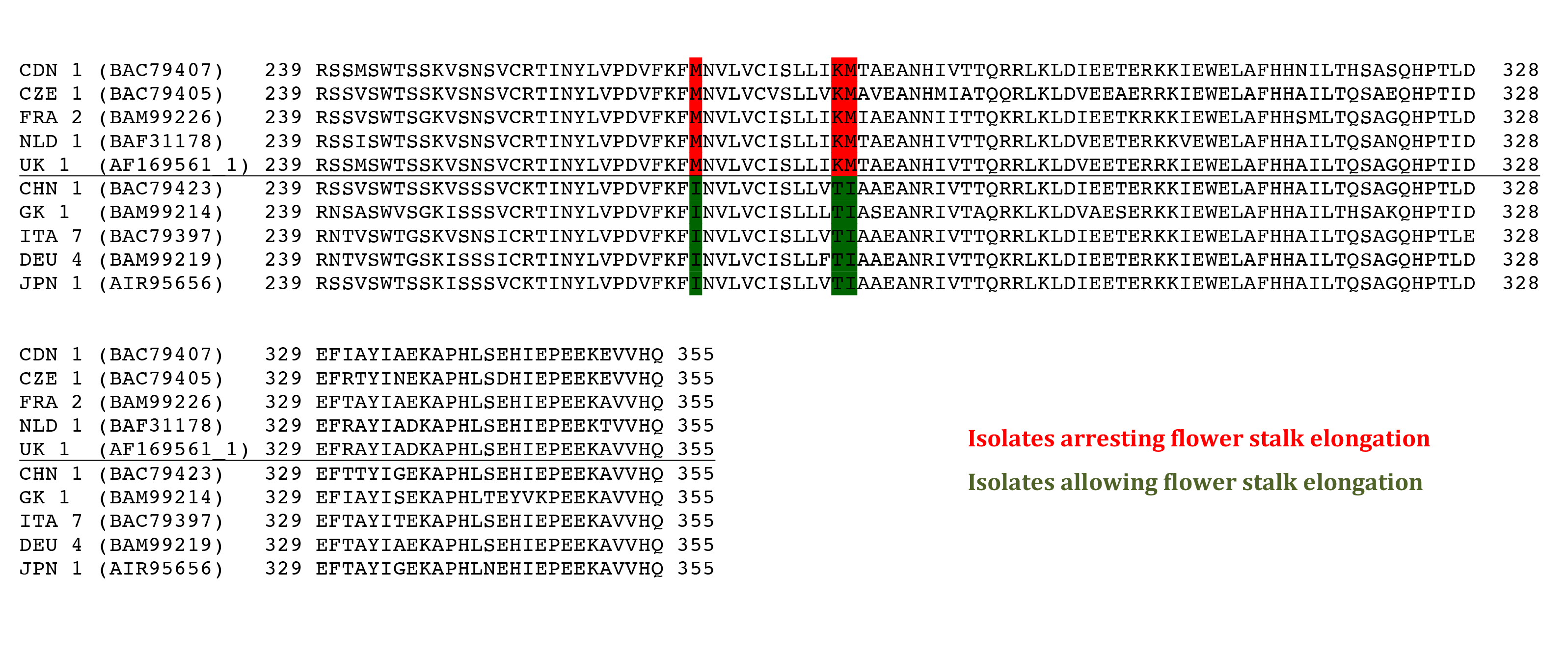

Supplement: Supplementary file 6 — Supplementary Material [file MPP-21-1271-s006.TIF]

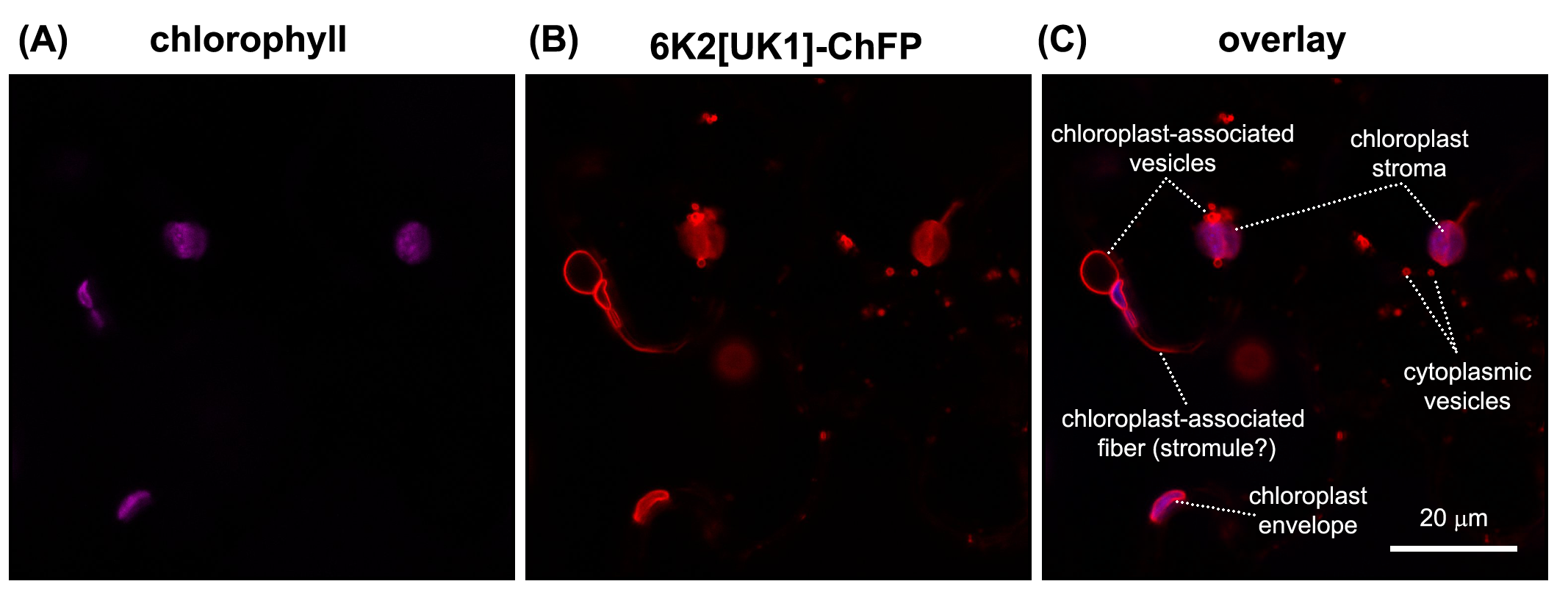

Supplement: Supplementary file 7 — Supplementary Material [file MPP-21-1271-s007.TIF]

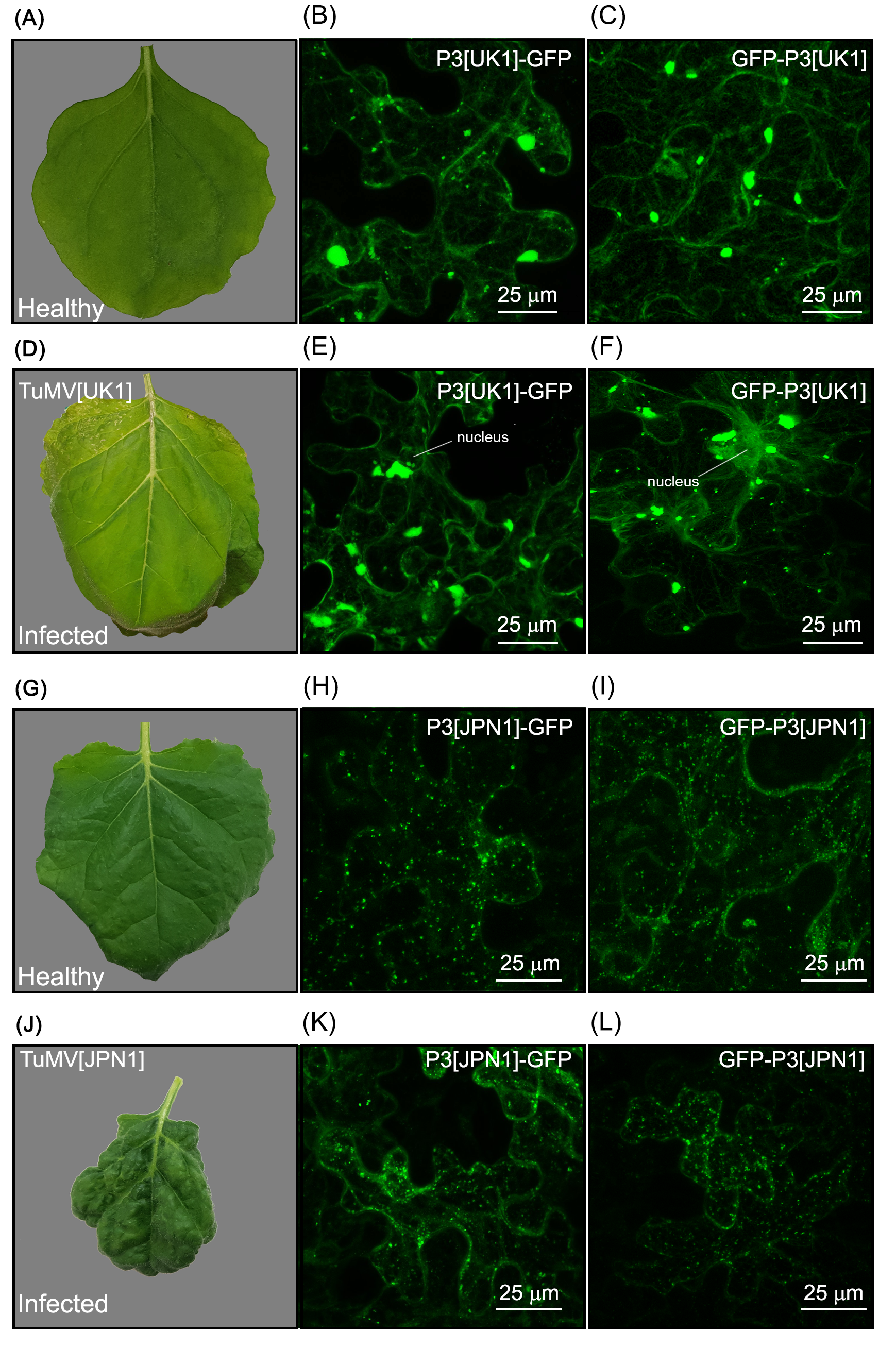

Supplement: Supplementary file 8 — Supplementary Material [file MPP-21-1271-s008.tif]

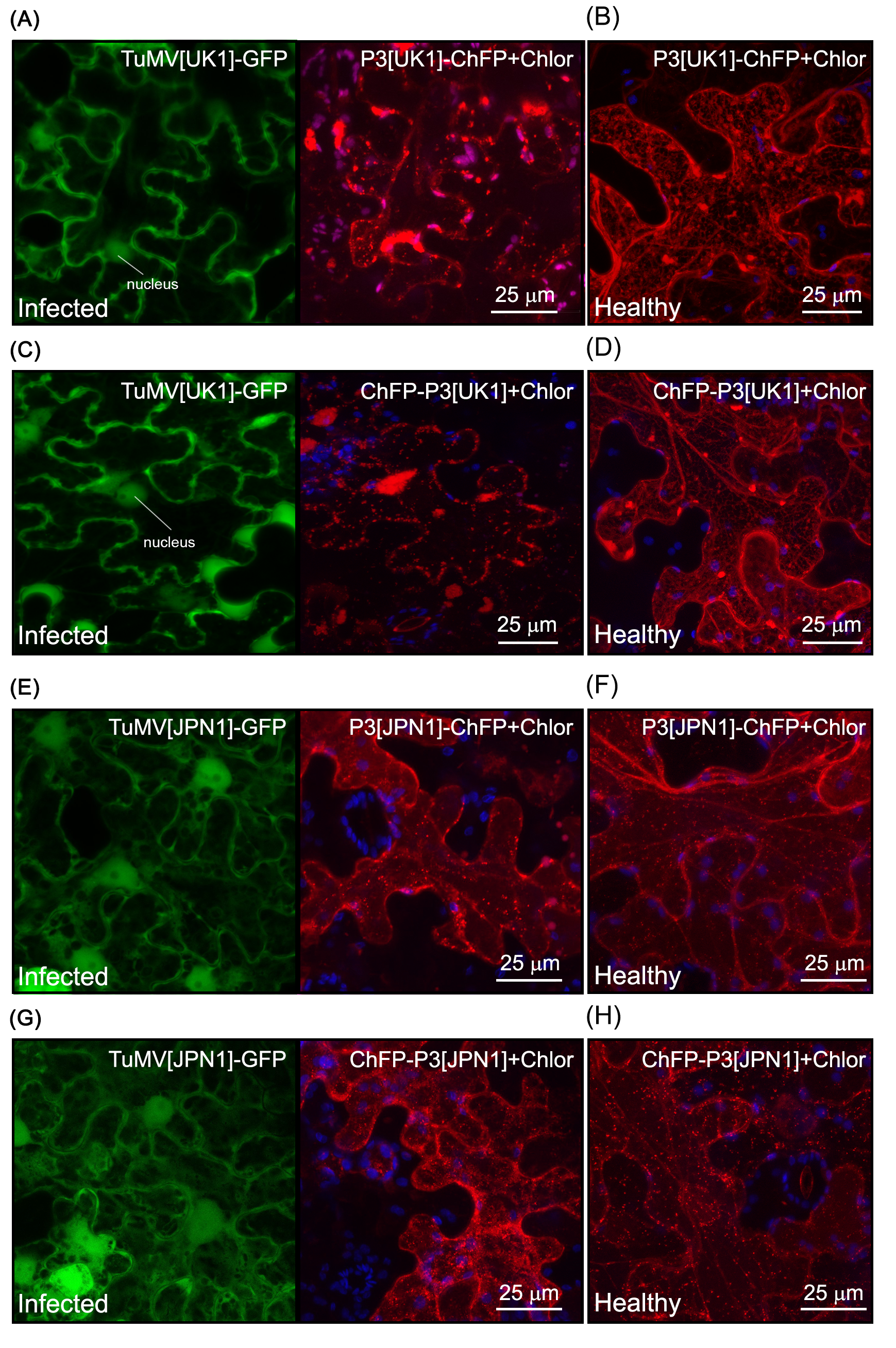

Supplement: Supplementary file 9 — Supplementary Material [file MPP-21-1271-s009.tif]
