# Supplementary material for: Association between flower stalk elongation, an Arabidopsis developmental trait, and the subcellular location and movement dynamics of the nonstructural protein P3 of Turnip mosaic virus
Source: Mol Plant Pathol. 2020 Aug 1;21(10):1271–86. doi: 10.1111/mpp.12976 (PMC7488469; doi:10.1111/mpp.12976)
Supplement: Supplementary file 10 — Supplementary Material [file MPP-21-1271-s010.DOCX]

**Table S1.** List of primers used in this work

| **Primer name** | **Primer sequence (5’-3’)** |
| --- | --- |
| P3[UK1]-(F) | ACGTCCATGGGAACAGAATGGGAGGACAC |
| P3[UK1]-(R) | ACGTGCTAGCTTGATGAACCACCGCCTTTTC |
| P3[JPN1]-(F) | ACGTCCATGGGCACCGCGTGGGAAGGAGCACATGGGTTTAATAAC |
| P3[JPN1]-(R) | ACGTGCTAGCTTGGTGCACTACTGCTTCTCCTCAGGTTCAATATG |
| P3[UK1 K297T]-(F) | TTGTATCAGCTTACTAATCACGATGACTGCCGAGGCGAATC |
| P3[UK1 K297T]-(R) | GATTCGCCTCGGCAGTCATCGTGATTAGTAAGCTGATACAA |
| P3[JPN1 T297k]-(F) | TTGCATTAGTTTATTAGTCAAAATAGCTGCTGAAGCAAATC |
| P3[JPN1 T297k]-(R) | GATTGCTTCAGCAGCTATTTTGACTAATAAACTAATGCAA |
| 6K2[UK1]-(F) | ACGTCCATGGGCAACACCAGCGACATGAGCAAA |
| 6K2[UK1]-(R) | ACGTGCTAGCTTCATGGGTTACGGGTTC |
| 6K2[JPN1]-(F) | ACGTCCATGGGCAACACCAACGACATGAGCAAG |
| 6K2[JPN1]-(R) | ACGTGCTAGCCTCGTGGGTCACGGGTTC |
| ChFP-KDEL Sec (F) | ACGTCCATGGCCATGAAGACTAATCTTTTTC |
| ChFP-KDEL Sec (R) | ACGTGCTAGCACTGAATTCGGCCGAGGATAATG |
| TuMV[UK1] E1 (F) | AAGCGTGCACTCTAGTATAGGTAATAG |
| TuMV[UK1] E2 (R) | TGTGCTATTTTACCCGCGAC |
| TuMV[UK1 M268I] I1 (F) | CGAGTACATTGATAAACTTGAACACATC |
| TuMV[UK1 M268I] I2 (R) | GATGTGTTCAAGTTTATCAATGTACTCG |
| TuMV [UK1 K297T] I1 (F) | CGGCAGTCATCGTGATTAGTAAG |
| TuMV[UK1 K297T] I2 (R) | CTTACTAATCACGATGACTGCCG |
| TuMV[UK1 M280I] I1 (F) | CTCGGCAGTGATCTTGATTAGTAAG |
| TuMV[UK1 M280I] I2 (R) | CTTACTAATCAAGATCACTGCCGAG |
| TuMV[UK1 K297T-M280I] I1 (F) | CGGCAGTGATCGTGATTAGTAAG |
| TuMV[UK1 K297T-M280I] I2 (R) | CTTACTAATCACGATCACTGCCG |
| TuMV[JPN1] E1 (F) | TCGCCAACGCTGAATTGC |
| TuMV[JPN1] E2 (R) | CTGGAGAATTTCACCATCACTAC |
| TuMV[JPN1 I268M] I2 (F) | GTTTATTAGTCACAATGGCTGCTG |
| TuMV[JPN1 I268M] I1 (R) | CAGCAGCCATTGTGACTAATAAAC |
| TuMV[JPN1 T297k]I2 (F) | GTTTATTAGTCAAAATAGCTGCTG |
| TuMV[JPN1 T297k] I2 (R) | CAGCAGCTATTTTGACTAATAAAC |
| TuMV[JPN1 T297K-I280M] I2 (F) | GTTTATTAGTCAAAATGGCTGCTG |
| TuMV[JPN1 T297K-I280M] I1 (R) | CAGCAGCCATTTTGACTAATAAAC |

*Letters in parenthesis indicates Forward (F) and Reverse (R)

** Letters underlined indicates point mutations
